# Cell integrity limits ploidy in budding yeast

**DOI:** 10.1093/g3journal/jkae286

**Published:** 2025-01-13

**Authors:** Juliet Barker, Andrew Murray, Stephen P Bell

**Affiliations:** Department of Biology, Howard Hughes Medical Institute, Massachusetts Institute of Technology, Cambridge, MA 02139, USA; Department of Molecular and Cellular Biology, Harvard University, Cambridge, MA 02138, USA; Department of Biology, Howard Hughes Medical Institute, Massachusetts Institute of Technology, Cambridge, MA 02139, USA

**Keywords:** polyploidy, genome doubling, cell surface stress, cell cycle, cell size

## Abstract

Evidence suggests that increases in ploidy have occurred frequently in the evolutionary history of organisms and can serve adaptive functions to specialized somatic cells in multicellular organisms. However, the sudden multiplication of all chromosome content may present physiological challenges to the cells in which it occurs. Experimental studies have associated increases in ploidy with reduced cell survival and proliferation. To understand the physiological challenges that suddenly increased chromosome content imposes on cells, we used *S. cerevisiae* to ask how much chromosomal DNA cells may contain and what determines this limit. We generated polyploid cells using 2 distinct methods causing cells to undergo endoreplication and identified the maximum ploidy of these cells, 32–64C. We found that physical determinants that alleviate or exacerbate cell surface stress increase and decrease the limit to ploidy, respectively. We also used these cells to investigate gene expression changes associated with increased ploidy and identified the repression of genes involved in ergosterol biosynthesis. We propose that ploidy is inherently limited by the impacts of growth in size, which accompany whole-genome duplication, to cell surface integrity.

## Introduction

Great diversity exists in the amount of genetic material that cells contain among organisms across life’s domains (Cavalier-Smith 1982). Further, individuals from the same species, and cells from the same individual, can vary greatly in their amount of genetic material, including their ploidy (Fankhauser 1945; Mortimer 1957; Swarup 1959; Orr-Weaver 2015; Robinson *et al*. 2018; Small *et al*. 2021). Much of this diversity arises as a result of polyploidization, the multiplication of whole chromosomal content within a cell.

Although rules for determining the optimal ploidy for a cell type are unknown, evidence suggests that a cell’s ploidy is subject to evolutionary pressures. For example, polyploidy is prevalent in both organismal and cancer evolution (Bielski *et al*. 2018; reviewed in Zack *et al*. 2013; Van de Peer *et al*. 2017). Because cells that polyploidize are typically impeded in their ability to survive and proliferate (Ramsey and Schemske 1998; Andalis *et al*. 2004; Fujiwara *et al*. 2005; Fox *et al*. 2010), the persistence of polyploid organisms and cells suggests that polyploidy can confer adaptive evolutionary advantages in surviving progeny. In addition, endopolyploidy, which arises from cell cycles in which genome duplication is not followed by cell division, is essential to the development of some multicellular organisms (Edgar and Orr-Weaver 2001). In these cases, endopolyploidy appears to serve as an adaptation in cells with specialized functions that require increased cell size, gene expression, or metabolic rate (reviewed in Orr-Weaver 2015).

Ploidy consistently correlates with and has been proposed to determine cell size (Cavalier-Smith 1982). An association between cell size and ploidy has been observed in budding (Mortimer 1957) and fission yeast (Neumann and Nurse 2007), angiosperms (Jovtchev *et al*. 2006; Robinson *et al*. 2018), and many vertebrates (Fankhauser 1939; Swarup 1959; Henery and Kaufman 1992; Ganem *et al.* 2014; Gibeaux *et al*. 2018; Small *et al*. 2021). This widely shared correlation between ploidy and cell size suggests that genomic DNA imposes a minimum cell size. Further, when *S. cerevisiae* cells grow without DNA replication, they exhibit defects in gene expression, intracellular signaling, and cell cycle progression that are alleviated by increasing their ploidy (Neurohr *et al*. 2019). Together, these observations suggest that the amount of DNA within cells affects their form and physiology—and further that cells may contain too little DNA for their size, to the detriment of their physiology.

In this study, we used *S. cerevisiae* to ask the opposite question: what happens when cells undergo multiple rounds of DNA replication without intervening mitoses and cell divisions? We used 2 methods to alter the cell cycle so that successive rounds of DNA replication continually increase ploidy. We used these cells to address 2 questions: what is the maximum ploidy of *S. cerevisiae* cells and what determines this limit? We identify the range of ploidies that cells can reach, and demonstrate that reducing cell surface stress increases the maximum ploidy.

## Materials and methods

### Yeast strains

All yeast strains used in this study were derived from a W303 base strain. All strains and their genotypes and source are listed in Supplementary Table 3. PCR products were used for gene deletions and replacements as described in Longtine *et al*. (1998), and transformations were carried out by lithium acetate heat shock method (Schiestl and Gietz 1989). Transformed strains were mated and sporulated and tetrads dissected by micromanipulation to obtain desired genotypes.

### Yeast culture conditions

Cells were grown in yeast extract and peptone supplemented with adenine (0.055 mg/mL) and either 2% dextrose (YPD), 2% dextrose and 1 M sorbitol (YPD + sorbitol), or 2% dextrose and 0.5 M NaCl (YPD + NaCl). All time-course experiments were initiated with cells growing in log phase (0.2 < OD_600_ < 0.8) in their respective media and diluted to OD_600_ of 0.2 in equivalent, sterile media. All cultures of cells were maintained in log-phase growth throughout the duration of time-course experiments by diluting the culture with sterile medium. This sterile medium contained 15 μg/mL nocodazole when added to *mad1*Δ*  bub2*Δ cells. Cell culture was conducted at 30°C for *mad1Δ bub2Δ* cells and 24°C for exponentially growing, haploid *cdc24-1  swe1Δ* cells. To induce genome duplication in *cdc24-1  swe1Δ* cells, cells were cultured at 37°C in a shaking water bath.

### Induction of genome duplication

To induce genome duplication in *mad1Δ bub2Δ* cells, the cells were treated with 15 μg/mL nocodazole (Millipore Sigma) at the initiation of each experiment. Nocodazole was dissolved to a concentration of 10 mg/mL in DMSO (Millipore Sigma). To prevent the effects of cells’ adaptation to the presence of nocodazole, an additional 7.5 μg/mL of nocodazole was added to cultures every 4 h. Volume equivalents of DMSO were added to all exponentially growing *mad1Δ bub2Δ* control cultures. To induce genome duplication in *cdc24-1  swe1Δ* cells, the cells were added to flasks prewarmed to 37°C in a shaking water bath at the initiation of each experiment and grown at 37°C for the durations of the experiments. Exponentially growing *cdc24-1  swe1Δ* control cultures were maintained at 24°C in a shaker with air cooling.

### Antifungal drug treatments

To determine appropriate concentrations of antifungal drugs to treat endomitotic cells for our desired experiments, *mad1Δ bub2Δ* cells were grown in a SpectraMax iD5 microplate reader (Molecular Devices) in the presence of varying concentrations of the drugs with continuous orbital shaking and OD_600_ measurements every 10 min, in the absence of nocodazole, at 30°C. For *cdc24-1  swe1Δ* cells, the same conditions were used, except at room temperature. To determine the effects of antifungal drug treatment on endomitotic cells, the cells were treated with 8 μg/mL fluconazole (Millipore Sigma), 0.44 μg/mL terbinafine (Thermo Fisher), 0.3 μg/mL amorolfine (ApexBio), and 24.7 ng/mL amphotericin B (Millipore Sigma), in cell culture flasks.

### Cell volume determination

All cell volumes were determined by volume displacement using a Multisizer 3 (Beckman Coulter) and ISOTON II electrolyte (Beckman Coulter).

### Flow cytometry

All flow cytometry was conducted using a CytoFLEX flow cytometer (Beckman Coulter) and CytExpert acquisition software (Beckman Coulter). 50,000 cells were analyzed for all samples. FlowJo v10.7.2 (Becton Dickinson) was used to plot and conduct statistical analysis of all flow cytometry data.

### Quantification of cell viability

Cell staining for quantification of viability was performed as follows. Cells were collected by centrifugation, resuspended in solution containing 0.2 μM SYTOX Green Nucleic Acid Stain (Thermo Fisher Scientific), and incubated for 10 min, protected from light, before analysis by flow cytometry. SYTOX green intensity was measured using a 488-nm laser and 525/40 (FITC) band-pass filter. For cells grown in YPD, this solution was 50 mM sodium citrate. For cells grown in YPD + 1 M sorbitol, this solution was 1 M sorbitol, and for cells grown in YPD + 0.5 M NaCl, this solution was 0.5 M NaCl. Parameters and thresholds used to quantitate cell viability are depicted in Supplementary Fig. 8.

### Quantification of DNA content

Preparation of cells for DNA content analysis was performed as follows. Cells were fixed in 70% ethanol at −20°C overnight. Cells were collected by centrifugation, resuspended in 50 mM sodium citrate containing 250 mg/mL ribonuclease A (Thermo Fisher Scientific), and incubated at 37°C overnight. Cells were collected by centrifugation, resuspended in 50 mM sodium citrate containing 2 μM SYTOX Green Nucleic Acid Stain (Thermo Fisher Scientific), and incubated at room temperature for 2 h, protected from light. Cells were briefly sonicated to reduce clumps and analyzed by flow cytometry. SYTOX green intensity was measured using a 488 nm laser and 525/40 (FITC) band-pass filter. To determine the percentage of cells in samples displaying DNA contents of interest (1C, 2C, 4C, 8C, 16C, 32C), cells were binned and the frequency of cells in each bin was quantified. Since mean FITC values vary slightly in each experiment, the values delineating each bin were chosen uniquely for each experiment and kept constant for all samples in the same experiment. Across experiments, the values delineating each bin were chosen to correspond to local minima nearest the following values: FITC pulse area = 33–55 [arbitrary units (AU); 1C bin], FITC pulse area = 55–120 (AU; 2C bin), FITC pulse area = 120–235 (AU; 4C bin), FITC pulse area = 235–450 (AU; 8C bin), FITC pulse area = 450–890 (AU; 16C bin), and FITC pulse area = 890–2,700 (AU; 32C bin).

### Live-cell microscopy

Live-cell microscopy was conducted on a Delta Vision ultra-widefield fluorescence microscope (GE Healthcare) with a 60× 1.42 NA objective lens (Olympus), sCMOS camera, and temperature control chamber. Acquire Ultra software (GE Healthcare) was used for image acquisition, and SoftWoRx software (GE Healthcare) was used for image deconvolution. *mad1Δ bub2Δ* cells were mounted onto pads of YPD agar containing 30 μg/mL nocodazole and imaged, intermittently, at 30°C. *cdc24-1  swe1Δ* cells were mounted onto 2X synthetic complete (SC) medium (“SC Medium 2016”) agar pads and imaged, intermittently, at 37°C.

### Quantitative transcriptomics

#### RNA extraction

Cells were collected for RNA extraction by centrifugation, resuspended in diethyl pyrocarbonate-treated water, and immediately flash-frozen in liquid nitrogen. Total RNA was extracted from cells using an RNEasy Mini kit (QIAGEN) and the manufacturer's protocol for RNA extraction from yeast with the following specifications. Cells were mechanically lysed by bead milling using a FastPrep-24 5G instrument (MP Biomedicals). Samples contained between 0.5×10^6^ and 2×10^7^ cells, 600 μL of acid-washed glass beads (Sigma-Aldrich) and 600 μL lysis buffer (with β-mercaptoethanol), in 2-mL tubes, and were agitated for 2–8 rounds of 60 s at 6.5 m/s, with chilling on ice for 5-min intervening rounds, until cells were completely lysed. Complete lysis was confirmed by microscopy. The concentration and integrity of the extracted total RNA were determined using a 2100 BioAnalyzer (Agilent).

#### cDNA library generation and sequencing

mRNA was isolated and fragmented using an NEBNext Poly(A) mRNA Magnetic Isolation Module (New England BioLabs), and cDNA was synthesized, ligated to adaptors, and PCR-amplified for sequencing using an NEBNext Ultra II RNA Library Prep Kit for Illumina (New England BioLabs). Sequencing was conducted on NovaSeq6000 using an S4 flow cell (Illumina).

#### Sequencing data analysis

RNA-seq analysis was conducted using the nf-core/rnaseq pipeline (Ewels *et al*. 2020) with STAR for genome alignment and Salmon for transcript quantification (Patro *et al.* 2017). The *S. cerevisiae* S288c R64-1-1 assembly was used for the reference genome, sourced from the Saccharomyces Genome Database (Engel *et al.* 2022).

#### Differential expression testing

DESeq2 (Love *et al*. 2014) was used to test for differential gene expression in the condition of increased ploidy. Two independent tests of differential expression were conducted, each including 3 types of cells. One test included: (1) (polyploid) *mad1Δ bub2*Δ cells treated with nocodazole, (2) (diploid, arrested) *MAD1  BUB2* cells treated with nocodazole, and (3) (haploid, cycling) *mad1Δ bub2*Δ cells not treated with drug. The other test included: (1) (polyploid) *cdc24-1  swe1*Δ cells grown at 37°C, (2) (diploid, arrested) *cdc24-1  swe1*Δ, *cdc20-1* cells grown at 37°C, and (3) (haploid, cycling) *cdc24-1  swe1*Δ cells grown at 25°C. Each aforementioned cell type was represented with samples from the same culture at 0 h (experiment initiation), 4, 8, 12, and 16 h—except for haploid, cycling cells for which only 2 time points were represented, 0 and 16 h. For both tests, 3 biological replicate samples were included. To each individual sample subjected to RNA-seq, a mean DNA content value was assigned. To calculate this value, the DNA content of 50,000 cells (sampled at the same time and from the same culture as those cells used for RNA-seq) was first measured. Next, particles were removed before calculating the mean DNA content of those remaining, in 2 ways. First, the ratio of FITC pulse area to FITC pulse height was calculated and particles for which this value was >1.39 were discarded to remove cell doublets. Next, particles with a FITC pulse area <30 (AU) were discarded, to remove debris below 1C DNA content. From all remaining particles, the mean FITC pulse area (DNA content) value was calculated (Supplementary Fig. 3). The continuous variable DNA content was used to calculate the log_2_ fold change in each gene, per unit DNA content, and generate a Wald test *P*-value, using DESeq2.

#### Other statistical analysis

Gene Ontology (GO) Term Finder (version 0.86) from the Saccharomyces Genome Database was used to test for the enrichment of genes assigned to common GO terms. The list of 51 genes whose change in expression was considered significant in both *mad1Δ bub2*Δ and *cdc24-1  swe1*Δ polyploid cells was queried, and GO terms for which corrected *P*-value < 0.05, and false-discovery-rate < 10%, were considered statistically significant.

## Results

### Manipulating the cell cycle to assess maximal ploidy

**Fig. 1.**
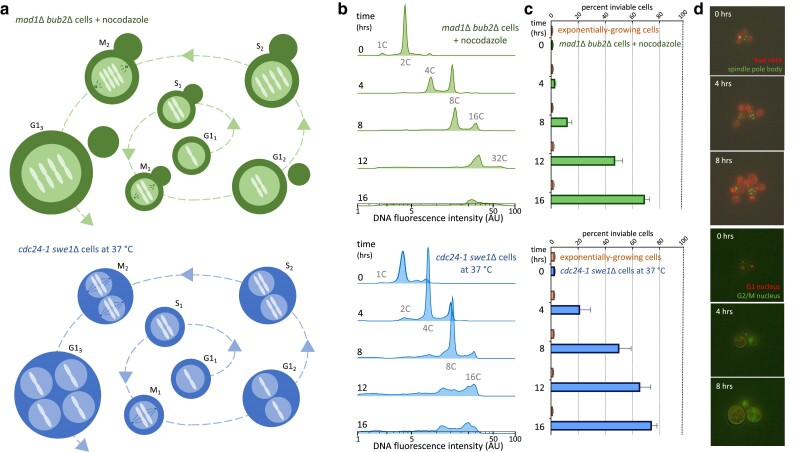
The maximal ploidy of *S. cerevisiae* is 16C–32C. a) Schematic of 2 cell types modified to undergo whole-genome duplication through endomitosis. b) DNA content during growth of endomitotic cells. Representative single experiments are shown. Additional replicate experiments and DNA content of exponentially growing control cells are included in Supplementary Fig. 5. c) Cell inviability, in cells undergoing endomitosis, and in exponentially growing control cells. Values are the mean of 3 experiments and were determined using a membrane-impermeant, fluorescent, nucleic acid stain. d) Fluorescence microscopy of live, endomitotic cells. *mad1Δ bub2*Δ cells express Cdc3-mCherry and Spc42-GFP fusion proteins, marking the bud neck and spindle pole bodies, respectively. *cdc24-1  swe1Δ* cells express a Whi5-mCherry and Clb2-GFP fusion proteins, marking the nucleus in G1 and G2/M phases, respectively.

To determine the maximum ploidy that *S. cerevisiae* cells can reach, we generated cells that increase in ploidy by undergoing endomitosis, DNA replication followed by incomplete mitosis (Fig. 1a). We postulated that by measuring the maximal ploidy of cells in which the accumulation of genomic DNA is achieved by distinct means, we could identify the maximum genomic DNA content.

To induce genome duplication without nuclear division, we inactivated the checkpoints that monitor the assembly and positioning of the mitotic spindle. We deleted *MAD1*, which is required for the spindle checkpoint (Li and Murray 1991), and *BUB2*, which is required for the spindle position checkpoint (Bloecher *et al*. 2000; Pereira *et al*. 2000). We treated the cells lacking these checkpoints with nocodazole, which depolymerizes microtubules and prevents spindle formation. Under these conditions, chromosomes do not segregate and the cell cycle arrest normally imposed by the spindle and spindle position checkpoints does not occur. In each cell cycle, the mother cell retains the entire duplicated genome in its nucleus, resulting in increasingly polyploid mother cells and daughter cells lacking DNA.

To induce genome duplication without cell division, we used cells carrying a temperature-sensitive allele of *CDC24* and lacking *SWE1*. *CDC24* is essential for bud formation (Hartwell *et al.* 1973) and *SWE1* inhibits cell cycle progression into mitosis in the absence of bud formation (Sia *et al*. 1996). When grown at the restrictive temperature, *cdc24-1  swe1*Δ cells do not bud and do not undergo the nuclear cycle delay normally imposed by *SWE1* in the absence of a bud. Although chromosome segregation and nuclear division occur within the nuclei of these nondividing cells, the replicated genomes are retained in the mother cell, resulting in complete genome duplication in each cell cycle.

By measuring DNA content over several generation times, we found that both cell types reach a common maximal ploidy of 16C–32C after 12 h of growth (Fig. 1b, Supplementary Fig. 5). Although most cells do not exceed 16C in DNA content, 16.2% (range 11.2–19.2%) of *mad1Δ bub2*Δ cells and 0.4% (range 0.1–0.7%) of *cdc24-1  swe1*Δ cells display a DNA content of greater than 16C, with none exceeding 32C (Fig. 1b). Cells allowed to grow for an additional 4 h did not show further increases in ploidy (Fig. 1b; 16 h). We conclude that 16C–32C is the maximal DNA content in the 2 conditions we tested.

Although many cells reach 16C, many cells in the populations containing these cells are inviable (Fig. 1c). Based on the entry of a membrane-impermeant dye, the fraction of inviable cells increases continuously with DNA content in each strain. In controls that do not increase in ploidy, achieved either by omitting nocodazole (*mad1Δ bub2*Δ cells) or growing cells at a permissive temperature (*cdc24-1  swe1*Δ cells), cell viability remains high and constant over the same experimental duration (Fig. 1c). We conclude that increased ploidy due to endomitosis reduces cell viability.

### Polyploidy is associated with changes in gene expression

What limits the maximal ploidy of endomitotic cells? We characterized the changes in gene expression accompanying increased ploidy using RNA-seq. We hypothesized that gene expression changes correlated with increased ploidy could identify the cellular structure or physiology that limits maximal DNA content. Such changes could reflect causes of cell inviability at maximal ploidy, or adaptive responses that maintain cell viability at maximal ploidy, for example.

To identify a gene expression signature characteristic of increased ploidy, we quantified transcripts from endomitotic cells sampled every 4 h, over a total of 16 h. In parallel, we measured the DNA content of the cells at each sampling (Supplementary Fig. 3). From these measurements, we identified genes whose expression changes significantly with the continuous variation in DNA content that occurs in endomitotic cells as they reach maximal ploidy.

We assessed gene expression in additional cells to disentangle changes in gene expression induced directly by polyploidy from those induced by the increase in cell size. Specifically, we measured gene expression in cells that grew in size without increasing in ploidy. To compare with polyploid *mad1Δ bub2*Δ cells, we treated cells with intact checkpoints (*MAD1  BUB2*) with nocodazole, causing them to arrest prior to anaphase with 2C DNA content (Dustin 1984). To compare with polyploid *cdc24-1  swe1*Δ cells, we introduced a temperature-sensitive allele of *CDC20, cdc20-1,* into *cdc24-1  swe1*Δ cells. At restrictive temperature, these cells arrest in mitosis with 2C DNA content because they cannot activate the anaphase-promoting complex (Sethi *et al*. 1991). We compared the gene expression changes in these cells to those in endomitotic cells and to those in exponentially growing and dividing, haploid *mad1Δ bub2*Δ (not treated with nocodazole) and *cdc24-1  swe1*Δ (at 24°C) cells, over the course of 16 h. By doing so, we controlled for the effects of genotype, experimental treatment, and enlarged cell size in our experiment, allowing us to detect gene expression specific to increased ploidy.

**Fig. 2.**
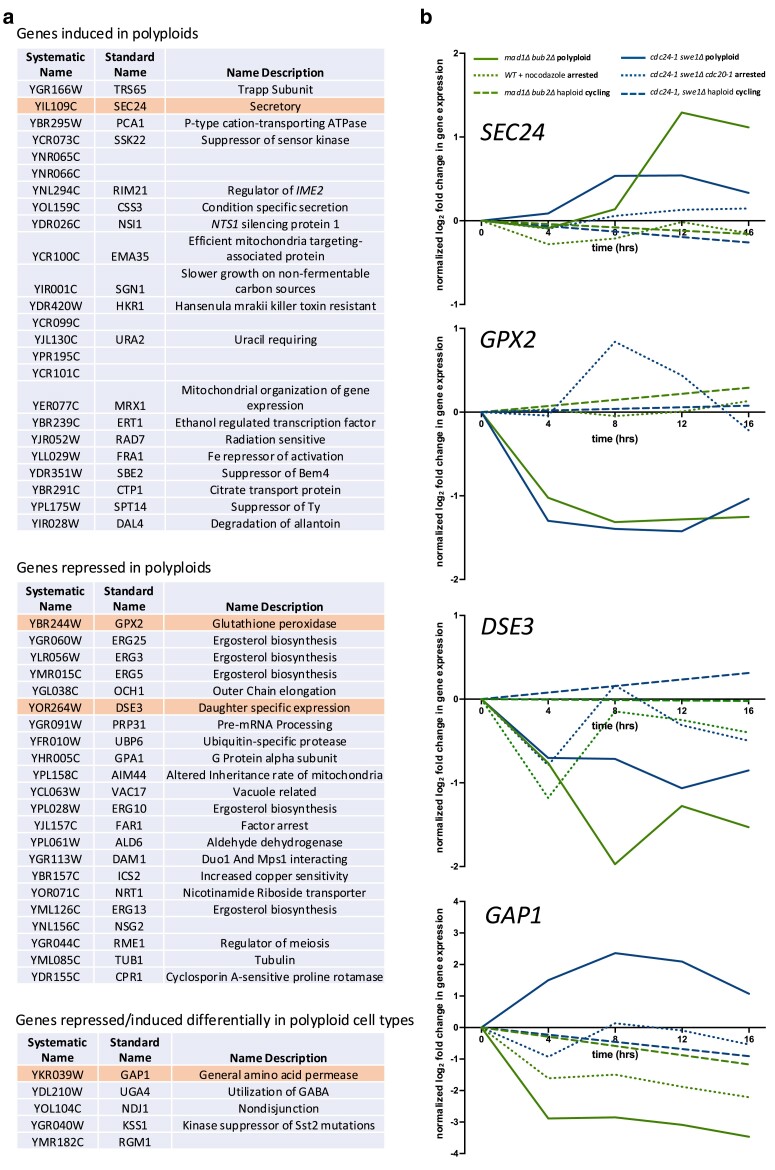
Gene expression signature characterizing polyploid cells. a) Genes differentially expressed in *mad1Δ bub2Δ* and *cdc24-1  swe1Δ* polyploid cells relative to their cell cycle-arrested and exponentially growing counterparts. Genes listed meet a significance level of *α* < 0.05 with Bonferroni’s correction in tests in both *mad1Δ bub2Δ* and *cdc24-1  swe1Δ* cells. Genes are distinguished by those induced in both polyploid cell types, repressed in both polyploid cells types, and induced in 1 polyploid cell type and repressed in the other. In each list, genes are ordered by ascending *P*-value, using the larger probability from the 2 tests (Supplementary Table 2, Supplementary Data). Highlighted genes appear in (b). b) Relative gene expression vs time of selected genes in *mad1Δ bub2Δ* and *cdc24-1  swe1Δ* cells. Gene expression levels are normalized to their values at experiment initiation. Lines represent the mean of 3 experimental replicates. Gene expression values were measured only at the beginning and end of the experiment (0 and 16 h) for haploid, cycling cells. Plots of additional genes and individual replicates are shown in Supplementary Fig. 9.

Using this comparison, we identified 51 genes that change in expression significantly in association with ploidy (Fig. 2a; Supplementary Table 2, Supplementary Data). The relative expression of selected genes with time since the induction of endomitosis is plotted in Fig. 2b and Supplementary Fig. 9. Forty-six of these genes are altered in expression in the same direction in both *mad1Δ bub2Δ* and *cdc24-1  swe1Δ* cells (e.g. Fig. 2b, *SEC24*). A further 5 genes were induced significantly in 1 polyploid cell type, but repressed in the other (e.g. Fig. 2b, *GAP1*). GO term enrichment analysis of the genes changed in expression with DNA content was significant (*P* < 0.01) only for terms associated with ergosterol biosynthesis (Supplementary Table 1). Genes involved in this process are repressed in polyploids generated by both methods. We conclude that significant gene expression changes are associated with increased ploidy and shared between *mad1Δ bub2Δ* and *cdc24-1  swe1Δ* cells.

To test whether cellular ergosterol biosynthesis impacts maximal ploidy, we treated endomitotic cells with drugs that target this pathway. We used concentrations of these drugs that modestly (<30%) increased the population doubling time of haploid, exponentially growing *mad1Δ bub2*Δ and *cdc24-1  swe1*Δ cells (Supplementary Fig. 4). Endomitotic cells grown in fluconazole, terbinafine, amorolfine, and amphotericin B did not exhibit differences in cell viability or maximal ploidy with respect to endomitotic cells not treated with these drugs (Supplementary Fig. 4). Thus, treatment with inhibitors of ergosterol synthesis or abundance did not increase the maximal ploidy of endomitotic cells. Although genetic methods to increase the presence of cellular ergosterol exist, these confer demonstrated pleiotropic phenotypes to cells (Crowley *et al*. 1998). Because we were not able to experimentally maintain or increase the presence of cellular ergosterol without confounding variables, we did not determine whether decreased ergosterol synthesis was required for cells to reach maximal ploidy, or raised their viability at increased ploidy.

### Increasing medium osmolarity increases endomitotic cell viability and maximal ploidy

Because the remaining genes identified in our analysis did not suggest a mechanism limiting maximal ploidy, we sought to determine what is responsible for this limit by other means. As ergosterol is essential to plasma membrane integrity and we observed that maximally polyploid cells die by lysing within hours of reaching their final ploidy (Fig. 1, Supplementary Fig. 10), we tested whether protecting cells from lysing affected the maximum ploidy we observed. We reasoned that if cell lysis imposed a limit to DNA content, then preventing it would increase this limit.

**Fig. 3.**
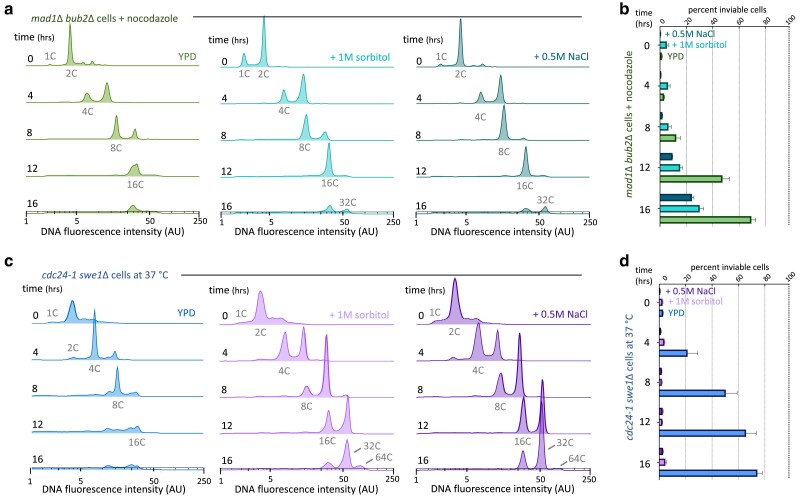
Maximal ploidy and viability of endomitotic cells are increased in high-osmolarity growth medium. a) DNA content of *mad1Δ bub2Δ* cells undergoing endomitosis in standard (described in Methods) and high-osmolarity media. b) Cell inviability of *mad1Δ bub2Δ* cells in standard and high-osmolarity media. c) DNA content of *cdc24-1  swe1Δ* cells undergoing endomitosis in standard and high-osmolarity media. d) Cell inviability of *cdc24-1  swe1Δ* cells in standard and high-osmolarity media. DNA content plots represent single experiments. Additional replicate experiments are included in Supplementary Fig. 6. Cell inviability values are the mean of 3 experiments.

Sorbitol is commonly used to prevent lysis of yeast cells with compromised cell walls, presumably by increasing extracellular osmolarity and reducing the turgor pressure exerted on cell membranes and walls (Hutchison and Hartwell 1967; Levin and Bartlett-Heubusch 1992; Irie *et al*. 1993; Lee *et al*. 1993). Consistent with our reasoning, adding 1 M sorbitol to growth media increased the maximal ploidy the cells reached (Fig. 3; Supplementary Fig. 6). These effects were most pronounced in *cdc24-1  swe1*Δ cells (Fig. 3c and d). When sorbitol was added to the growth media, 23.3% (range 20.1–28.7%) of all *mad1Δ bub2*Δ cells and 50.5% (range 49.6–52.2%) of all *cdc24-1  swe1*Δ cells displayed a DNA content of 32C after 16 h of growth (Fig. 3a and c). For the *mad1Δ bub2*Δ genotype, this represents a 1.4-fold increase, and for the *cdc24-1  swe1*Δ genotype, a >125-fold increase in the fraction of cells reaching 32C in DNA content. Furthermore, 12% (range 7.5–16.4%) of all *cdc24-1  swe1*Δ cells reached 64C, a DNA content that was only observed with increased extracellular osmolarity (Fig. 3c).

Measuring cell viability in conditions with increased osmolarity also supported our hypothesis. When *mad1Δ bub2*Δ cells were grown in the presence of sorbitol, a greater proportion were viable at time points from 8 to 16 h (Fig. 3b). The addition of sorbitol to growth media increased the viability of *cdc24-1  swe1*Δ cells even more dramatically and across all time points (Fig. 3d).

The effects of increased osmolarity were not specific to sorbitol. Osmotic support provided by adding 0.5 M sodium chloride to growth medium increased both maximal ploidy and cell viability similarly to sorbitol (Fig. 3). When sodium chloride was added to growth media, 32.0% (range 26.4–38.1%) of all *mad1Δ bub2*Δ cells and 47.8% (range 36.0–57.9%) of all *cdc24-1  swe1*Δ cells displayed a DNA content of 32C after 16 h of growth (Fig. 3a and c). Additionally, 10.4% (range 10.1–10.6%) of all *cdc24-1  swe1*Δ cells displayed a DNA content of 64C (Fig. 3c). For both *mad1Δ bub2*Δ and *cdc24-1  swe1*Δ cells, the addition of sodium chloride also increased the viable proportion of cells at all time points (Fig. 3b and d). We conclude that increased medium osmolarity increases maximal ploidy by improving endomitotic cell viability.

### Modifying cell size affects endomitotic cell viability and maximal ploidy itself

**Fig. 4.**
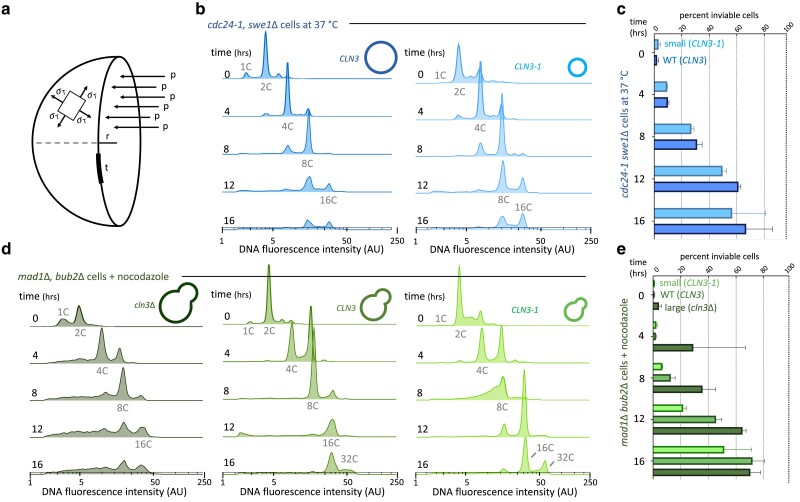
Maximal ploidy and viability of endomitotic cells are increased in cells of reduced size. a) Tangential stress (*σ_t_*) in the plane of a sphere’s surface. *P* = turgor pressure, *r* = cell radius, and *t* = surface thickness. b) DNA content of *cdc24-1  swe1Δ* cells with *CLN3* and *CLN3-1* undergoing endomitosis. c) Cell inviability of *cdc24-1  swe1Δ* cells. d) DNA content of *mad1Δ bub2Δ* cells with *cln3Δ*, *CLN3* and *CLN3-1* undergoing endomitosis. e) Cell inviability of *mad1Δ bub2Δ* cells. DNA content plots represent single experiments. Additional replicate experiments and DNA content of exponentially growing control cells are included in Supplementary Fig. 8. Cell inviability values are the mean of 3 experiments.

If higher external osmolarity increases maximal ploidy by reducing physical stress on the cell surface, reducing cell size should have a similar effect. Budding yeast cells are approximately spherical, with thin cell walls relative to their diameter. Hence, force in the plane of the cell’s surface per unit area (surface/tangential stress; *σ_t_*) is approximated by the equation *σ_t_ = pr*/2*t*, where *p* is the turgor pressure, *r* is the cell radius, and *t* is the cell surface thickness (Koch 1990; Fig. 4a). Cell surface stress is therefore directly proportionate to both turgor pressure and cell radius and will increase as polyploid cells grow in size. Since reducing turgor pressure in polyploid cells by increasing the osmolarity of the growth media increased their maximal ploidy and viability (Fig. 3), we hypothesized that reducing the cell radius would have similar effects.

To test this hypothesis, we created mutants of *mad1Δ bub2*Δ and *cdc24-1  swe1*Δ cells with altered mean cell volume and, therefore, cell radius. To do so, we introduced both hypo- and hypermorphic alleles of the G1 cyclin-encoding gene, *CLN3*, into both strains. Cells lacking *CLN3* have longer G1 durations and initiate S phase at larger sizes (Nash *et al*. 1988). Cells possessing hypermorphic alleles of *CLN3* have reduced G1 durations and initiate S phase at smaller sizes ( Tyers *et al.* 1992). We made hypermorphs (*CLN3-1*) of *CLN3* by truncating the gene to remove a domain of its encoded protein that reduces the protein’s lifetime. Haploid *cln3*Δ cells are 67% larger, and *CLN3-1* cells are 10–29% smaller than cells expressing wild-type *CLN3* (Supplementary Fig. 1). In both *mad1Δ bub2*Δ and *cdc24-1  swe1*Δ cells, *CLN3-1* cells remain smaller, and in *mad1Δ bub2*Δ cells, *cln3*Δ cells remain larger than *CLN3* cells during 12 h of endocycling (Supplementary Fig. 2).

In both *mad1Δ bub2*Δ and *cdc24-1  swe1*Δ cells, making cells smaller led to higher ploidy and making them bigger reduced their maximal ploidy (Fig. 4; Supplementary Fig. 7). In *mad1Δ bub2*Δ cells, 2-fold more *CLN3-1* cells reached 32C in ploidy relative to cells expressing *CLN3* (Fig. 4d), but only half as many of the enlarged *mad1Δ bub2*Δ *cln3*Δ cells reached 16C in ploidy compared with cells of wild-type size, and none reached 32C in ploidy (Fig. 4d; Supplementary Fig. 7). These effects in maximal ploidy were reflected in the relative proportion of viable, polyploid cells (Fig. 4e). At all experimental time points, small, *CLN3-1*, cells had the largest proportion of viable cells and large, *cln3Δ*, cells had the smallest (Fig. 4e).

We saw similar effects of reducing cell size in *cdc24-1  swe1*Δ cells (Fig. 4b and c). 20% more of *CLN3-1 cdc24-1  swe1*Δ cells reached a maximal ploidy of 16C and a slightly greater proportion of these cells were viable, compared with cells of wild-type size (Fig. 4b and c). We could not test the effect of enlarging *cdc24-1  swe1*Δ cells, as we found *cdc24-1, swe1*Δ, and *cln3*Δ to be synthetically lethal in our strains. We conclude that maximal ploidy and polyploid cell viability are inversely related to cell size, consistent with cell surface stress limiting maximal ploidy.

## Discussion

We used budding yeast as a model to ask what limits the ploidy that eukaryotic cells can reach. Using 2 independent methods to allow cells to repeatedly duplicate their genomes, we found that yeast cells reach a maximum DNA content of 32–64C. Reducing cell size and increasing medium osmolarity raise maximum ploidy, supporting the hypothesis that cell surface stress limits ploidy.

We also identified gene expression changes that characterize newly formed and highly polyploid cells. We attribute these gene expression changes to increased ploidy itself for 2 reasons. First, they are shared between cells altered in independent ways to become polyploid. Second, these changes were not observed in cells that have increased in size but not ploidy.

The most prominent change in gene expression we identified associated with increased ploidy was the repression of genes involved in ergosterol biosynthesis. Ergosterol is a component of cell membranes essential to their physical properties and function. The reduced abundance of cellular ergosterol (which is suggested by the repression of ergosterol biosynthesis genes) could serve to adapt cell membranes to increased stress, for example, and permit cells to reach greater ploidy. However, our experiments inhibiting ergosterol biosynthesis did not affect the maximum ploidy that cells attained, suggesting that ergosterol biosynthesis is not sufficient to affect the maximum ploidy of cells or that our treatments were insufficient to observe this effect.

Our study identified only 2 genes (GPA1 and NDJ1) in common with a previous study of gene expression changes associated with polyploidy (Wu *et al*. 2010). There are several differences between our study and this previous study that may explain the few genes identified in common. Our study identified gene expression changes associated with newly formed and successively increasing polyploidy, included cells of 8C, 16C, and 32C in ploidy, and incorporated controls for cell size. The study by Wu et al. identified expression changes characteristic of stably propagating tetraploid cells enlarged in size.

Reducing turgor pressure, by growing cells in hyperosmotic medium or reducing their size, increased the viability and maximal ploidy of endomitotic cells. Further, increasing cell surface stress by increasing cell size reduced cell viability and the fraction of cells that reach maximal ploidy. Taken together, our results support the hypothesis that reducing stress in the cell surface increases the viability of endomitotic cells, thus increasing their maximal ploidy. Our study did not address whether the surface of polyploid cells is compromised by an increased rate of cell growth (biomass added per unit of time) that may be associated with increased ploidy, absolute cell size, or a combination of both.

*Saccharomyces cerevisiae* possess an intracellular signaling pathway that senses and responds to cell wall compromise. Genetic mutations that both enhance and abolish the activity of factors required for this response have been characterized. Because, in our experience, these mutants independently and substantially impair cell viability, we could not rigorously test the contribution of this signaling pathway to the maintenance of polyploid cell viability or the limit to maximal ploidy.

Few examples exist in nature of cells that, by endoreplication, reach maximum degrees of ploidy (32–64C) near that of cells in this study (Orr-Weaver 2015). Two examples include subperineural glial cells of *Drosophila* (32C; Desalvo *et al*. 2011; Unhavaithaya and Orr-Weaver 2012) and giant neurons of slugs (10,000–200,000C; Lasek and Dower 1971; Yamagishi *et al*. 2012). These examples share in common characteristically flattened or elongated cell shapes distinct from budding yeast. The nearly spherical shape, and consequently low surface area-to-volume ratio, of *S. cerevisiae* may especially predispose these cells to stress on the cell surface, with increasing ploidy and size, that limits their viability.

## Supplementary Material

jkae286_Supplementary_Data

## Data Availability

Strains are available upon request. Gene expression data are available at GEO with the accession number: GSE275298. Detailed gene expression quantitation and statistics for all genes tested, including test statistics, are included in “Supplementary Data.” Supplementary Figures 1 and 2 contain cell volume measurements relevant to this study. Supplementary Figure 3 contains mean DNA contents assigned to each sample for the determination of gene expression changes associated with ploidy. Supplementary Figure 4 contains results of antifungal drug treatments on cell proliferation and endopolyploidy. Supplementary Figures 5–7 contain DNA content of cells from additional replicate experiments to those for which DNA content is depicted in Figs. 1, 3, and 4, respectively. Supplementary Figure 8 contains fluorescence intensity vs. forward scatter values from single, representative experiments used to determine cell viability and depicts gates used to quantitate the fraction of viable and inviable cells, and applied to all experiments. Supplementary Figure 9 contains plots of gene expression vs. time for selected genes whose expression changes significantly with ploidy. Supplementary Figure 10 contains live, fluorescence, time-lapse images of endocycling cells. Supplementary Table 1 contains results of GO term enrichment analysis of genes differentially expressed with polyploidy. Supplementary Table 2 contains all genes whose expression is changed significantly with ploidy (*α* < 0.05 after Bonferroni’s correction), their direction of change in expression with ploidy, and associated *P*-values. Supplementary Table 3 contains all strains used in this study. Supplemental material are available at G3 online.
