# Supplementary material for: Cell integrity limits ploidy in budding yeast
Source: G3 (Bethesda). 2025 Jan 13;15(2):jkae286. doi: 10.1093/g3journal/jkae286 (PMC11797008; doi:10.1093/g3journal/jkae286)
Supplement: jkae286_Supplementary_Data [file jkae286_supplementary_data.zip › Supplementary_Legends_G3-2024-405275.docx]

**Supplementary Data Titles and Legends**

**Fig. S1. Example volumes of cells in this study. (A)** Volumes of endomitotic, *cdc24-1 swe1*Δ cells grown at 37°C. **(B)** Volumes of endomitotic, *mad1*Δ *bub2*Δ cells grown with nocodazole. **(C)** Volumes of haploid, *CLN3* and *CLN3-1, cdc24-1 swe1*Δ cells **(D)** Volumes of haploid, *CLN3*, *cln3Δ*, and *CLN3-1, mad1*Δ *bub2*Δ cells. **(C and D)** Mode cell volumes of each population of haploid cells are labeled in shades corresponding with genotype labels.

**Fig S2. Example volumes of endocycling cells with variant alleles of *CLN3*, with time. (A)** Volumes of endocycling *mad1*Δ *bub2*Δ cells (carrying *CLN3, CLN3-1*, and *cln3*Δ alleles) at times 0, 4, 8, 12, and 16 hours. **(B)** Volumes of endocycling *cdc24-1 swe1*Δ cells (carrying *CLN3,* and *CLN3-1* alleles) at times 0, 4, 8, 12, and 16 hours.

**Fig. S3** **Mean DNA content assigned to samples in the determination of gene expression changes associated with ploidy.** **(A)** *mad1*Δ *bub2*Δ (circles) and *MAD1 BUB2* cells (triangles). **(B)** *cdc24-1 swe1*Δ (circles) and *cdc24-1 cdc20-1 swe1*Δ (triangles) cells. Y-axis depicts mean DNA content (arbitrary units), from a sample of cells representing those used for gene expression analysis. Methods for determining these values are described in Materials and Methods - Differential expression testing.

**Fig. S4.** **Reducing cellular ergosterol with antifungal treatment does not affect maximal ploidy. (A)** Effect of chosen antifungal drugs and their concentrations on the proliferation of haploid cells. OD_600_ versus time for *mad1*Δ *bub2*Δ cells (circles; grown in the absence of nocodazole) and *cdc24-1* *swe1*Δ cells (squares; grown at room temperature) in the presence of antifungal drugs. Yellow lines represent the growth of cells in drug concentrations used in (B and C). **(B and C)** DNA content of endomitotic *mad1*Δ *bub2*Δ **(B)** and *cdc24-1* *swe1*Δ **(C)** cells in the absence and presence of antifungal drugs.

**Fig. S5. Additional experimental replicates (related to Fig. 1). (A)** DNA content over time of *mad1*Δ *bub2*Δ cells. **(B)** DNA content over time of *cdc24-1* *swe1*Δ cells.

**Fig. S6. Additional experimental replicates (related to Fig. 3).** **(A)** DNA content over time of *mad1*Δ *bub2*Δ cells, grown with nocodazole or DMSO, and 1M sorbitol or 0.5M NaCl. **(B)** DNA content over time of *cdc24-1* *swe1*Δ cells grown at 37°C or 25°C, and with 1M sorbitol or 0.5M NaCl.

**Fig. S7. Additional experimental replicates (related to Fig. 4).** DNA content over time of *cln3Δ, CLN3,* and *CLN3-1* endomitotic cells **(A)** Two additional experimental trials with *mad1*Δ *bub2*Δ cells grown in nocodazole. **(B)** Two additional experimental trials with *cdc24-1* *swe1*Δ cells grown at 37°C.

**Fig. S8. Thresholds used to determine cell viability.** All plots depict particle fluoresence intensity (AU; fluo) vs. forward scatter (AU; fwd sca). Particles are divided into 4 quadrants (clockwise from top left; Q1 = fluo +, fwd sca -; Q2 = fluo +, fwd sca +; Q3 = fluo -, fwd sca+; Q4 = fluo -, fwd sca-). For *mad1*Δ *bub2*Δ cells (left side), quadrant gates are set to remove anucleate cells that form during the experiment, before determining the fraction of inviable cells. For experiements with *mad1*Δ *bub2*Δ cells, the fraction of inviable cells = (# cells in Q2)/(# cells in Q2 + # cells in Q3). Thus, all particles in Q1 and Q4 are excluded from this calculation. Time = 0 is an exception, for which all quadrants are includd and the fraction of inviable cells = (# cells in Q1 + # cells in Q2)/(# cells in Q1 + # cells in Q2 + # cells in Q3 + # cells in Q4). For *cdc24*-1 *swe1*Δ cells, quadrant gates are set to distinguish only between fluo+ and fluo- cells. For all time points for these cells, Q1 and Q4 contain 0 cells, and the fraction of inviable cells = (# cells in Q2)/(# cells in Q2 + # cells in Q3). **(A)** Single, representative experiment comparing haploid *mad1*Δ *bub2*Δ cells (+DMSO) to endomitotic *mad1*Δ *bub2*Δ cells (+ nocodazole), and haploid *cdc24*-1 *swe1*Δ cells (25°C) and endomitotic *cdc24*-1 *swe1*Δ cells (37°C). **(B)** Single, representative experiment comparing enodmitotic cells grown with and without osmolytes added to growth medium. **(C)** Single, representative experiment comparing *cln3*Δ, *CLN3*, and *CLN3-1*, *mad1*Δ *bub2*Δ cells (+ nocodaozle) and comparing *CLN3* and *CLN3-1 cdc24*-1 *swe1*Δ cells (37°C).

**Figure S9. Gene expression versus time for selected genes. (A)** Common axes labels and legend for all plots in (B-D). **(B)** Normalized expression values (3 individual replicates) versus time for genes plotted in Fig. 10. **(C and D)** Normalized expression values (mean, above; individual replicates, below) versus time for additional genes, listed in Fig. 10. **(C)** Genes induced in polyploidy. **(D)** Genes repressed in polyploidy.

**Fig. S10. Endomitotic cells undergo lysis at high DNA content.** Fluorescence microscopy of endomitotic, *cdc24-1 swe1Δ* cells. Cells express Whi5-mCherry and Clb2-GFP fusion proteins, marking the nucleus in G1 and G2/M phase, respectively. Marked increase in fluorescence at 8 hours and later time points results from autofluorescence of lysed cells.

**Table S1. Significant Gene Ontology (GO) terms, describing the list of 51 genes differentially- expressed in polyploidy cells (Fig. 10; Table S2).** Go terms whose constituent genes are enriched in the polyploid expression signature with p < 0.01 are shown. FDR = false discovery rate.

**Table S2. Genes whose expression is changed significantly with ploidy.** “expr dir” = expression direction. “UP” = induced with increased ploidy. “DOWN” = repressed with increased ploidy. Only those genes whose p-values are significant (𝛼 < 0.05) after Bonferroni correction in tests in *mad1*Δ *bub2*Δ and *cdc24-1 swe1*Δ cells are included. p-values depicted do not reflect Bonferroni correction. Genes are ranked by the larger of their two p-values, from both tests (“maximum p-value”).

**Table S3.** Strains used in this study.
