# Supplementary material for: Cell integrity limits ploidy in budding yeast
Source: G3 (Bethesda). 2025 Jan 13;15(2):jkae286. doi: 10.1093/g3journal/jkae286 (PMC11797008; doi:10.1093/g3journal/jkae286)
Supplement: jkae286_Supplementary_Data [file jkae286_supplementary_data.zip › Table_S1_G3-2024-405275.docx]

| **Gene Ontology term** | **Corrected P-value** | **Genes annotated to the term** |
| --- | --- | --- |
| [sterol desaturase activity](https://www.yeastgenome.org/go/GO:0070704) | 0.00884 | [YMR015C](https://www.yeastgenome.org/locus/YMR015C), [YLR056W](https://www.yeastgenome.org/locus/YLR056W) |
| [steroid biosynthetic process](https://www.yeastgenome.org/go/GO:0006694) | 0.00122 | [YGR060W](https://www.yeastgenome.org/locus/YGR060W), [YML126C](https://www.yeastgenome.org/locus/YML126C), [YLR056W](https://www.yeastgenome.org/locus/YLR056W), [YPL028W](https://www.yeastgenome.org/locus/YPL028W), [YNL156C](https://www.yeastgenome.org/locus/YNL156C), [YMR015C](https://www.yeastgenome.org/locus/YMR015C) |
| [sterol biosynthetic process](https://www.yeastgenome.org/go/GO:0016126) | 0.00122 | [YNL156C](https://www.yeastgenome.org/locus/YNL156C), [YMR015C](https://www.yeastgenome.org/locus/YMR015C), [YLR056W](https://www.yeastgenome.org/locus/YLR056W), [YPL028W](https://www.yeastgenome.org/locus/YPL028W), [YML126C](https://www.yeastgenome.org/locus/YML126C), [YGR060W](https://www.yeastgenome.org/locus/YGR060W) |
| [sterol metabolic process](https://www.yeastgenome.org/go/GO:0016125) | 0.00682 | [YLR056W](https://www.yeastgenome.org/locus/YLR056W), [YPL028W](https://www.yeastgenome.org/locus/YPL028W), [YNL156C](https://www.yeastgenome.org/locus/YNL156C), [YMR015C](https://www.yeastgenome.org/locus/YMR015C), [YGR060W](https://www.yeastgenome.org/locus/YGR060W), [YML126C](https://www.yeastgenome.org/locus/YML126C) |
| [steroid metabolic process](https://www.yeastgenome.org/go/GO:0008202) | 0.00866 | [YLR056W](https://www.yeastgenome.org/locus/YLR056W), [YPL028W](https://www.yeastgenome.org/locus/YPL028W), [YNL156C](https://www.yeastgenome.org/locus/YNL156C), [YMR015C](https://www.yeastgenome.org/locus/YMR015C), [YGR060W](https://www.yeastgenome.org/locus/YGR060W), [YML126C](https://www.yeastgenome.org/locus/YML126C) |
| [ergosterol biosynthetic process](https://www.yeastgenome.org/go/GO:0006696) | 0.00948 | [YML126C](https://www.yeastgenome.org/locus/YML126C), [YMR015C](https://www.yeastgenome.org/locus/YMR015C), [YGR060W](https://www.yeastgenome.org/locus/YGR060W), [YLR056W](https://www.yeastgenome.org/locus/YLR056W), [YPL028W](https://www.yeastgenome.org/locus/YPL028W) |
| [phytosteroid biosynthetic process](https://www.yeastgenome.org/go/GO:0016129) | 0.00948 | [YPL028W](https://www.yeastgenome.org/locus/YPL028W), [YGR060W](https://www.yeastgenome.org/locus/YGR060W), [YLR056W](https://www.yeastgenome.org/locus/YLR056W), [YMR015C](https://www.yeastgenome.org/locus/YMR015C), [YML126C](https://www.yeastgenome.org/locus/YML126C) |
| [cellular lipid biosynthetic process](https://www.yeastgenome.org/go/GO:0097384) | 0.00948 | [YGR060W](https://www.yeastgenome.org/locus/YGR060W), [YLR056W](https://www.yeastgenome.org/locus/YLR056W), [YPL028W](https://www.yeastgenome.org/locus/YPL028W), [YML126C](https://www.yeastgenome.org/locus/YML126C), [YMR015C](https://www.yeastgenome.org/locus/YMR015C) |
