# Supplementary material for: Cell integrity limits ploidy in budding yeast
Source: G3 (Bethesda). 2025 Jan 13;15(2):jkae286. doi: 10.1093/g3journal/jkae286 (PMC11797008; doi:10.1093/g3journal/jkae286)
Supplement: jkae286_Supplementary_Data [file jkae286_supplementary_data.zip › Table_S2_G3-2024-405275.docx]

| **Gene (systematic)** | **Gene (standard)** | **Gene Name Description** | **mad1Δ bub2Δ expr dir** | **cdc24-1 swe1Δ expr dir** | **mad1Δ bub2Δ p-value** | **cdc24-1 swe1Δ p-value** | **maximum p-value** |
| --- | --- | --- | --- | --- | --- | --- | --- |
| YGR166W | TRS65 | TRapp Subunit | UP | UP | 3.7454E-34 | 4.66974E-18 | 4.66974E-18 |
| YIL109C | SEC24 | SECretory | UP | UP | 4.83716E-17 | 7.92284E-15 | 7.92284E-15 |
| YBR295W | PCA1 | P-type Cation-transporting ATPase | UP | UP | 2.18688E-14 | 5.59992E-14 | 5.59992E-14 |
| YBR244W | GPX2 | Glutathione PeroXidase | DOWN | DOWN | 2.03682E-12 | 7.77206E-16 | 2.03682E-12 |
| YGR060W | ERG25 | ERGosterol biosynthesis | DOWN | DOWN | 1.07641E-15 | 2.21285E-12 | 2.21285E-12 |
| YCR073C | SSK22 | Suppressor of Sensor Kinase | UP | UP | 2.88248E-12 | 1.79332E-19 | 2.88248E-12 |
| YLR056W | ERG3 | ERGosterol biosynthesis | DOWN | DOWN | 1.31747E-13 | 8.78474E-12 | 8.78474E-12 |
| YMR015C | ERG5 | ERGosterol biosynthesis | DOWN | DOWN | 1.01321E-11 | 2.25533E-12 | 1.01321E-11 |
| YGL038C | OCH1 | Outer CHain elongation | DOWN | DOWN | 1.01356E-16 | 2.24218E-11 | 2.24218E-11 |
| YOR264W | DSE3 | Daughter Specific Expression | DOWN | DOWN | 1.32248E-15 | 3.73883E-11 | 3.73883E-11 |
| YNR065C |  |  | UP | UP | 1.22534E-10 | 2.66095E-15 | 1.22534E-10 |
| YNR066C |  |  | UP | UP | 1.89631E-10 | 7.20078E-13 | 1.89631E-10 |
| YGR091W | PRP31 | Pre-mRNA Processing | DOWN | DOWN | 3.84317E-13 | 1.92527E-10 | 1.92527E-10 |
| YNL294C | RIM21 | Regulator of IME2 | UP | UP | 4.11683E-17 | 4.93379E-10 | 4.93379E-10 |
| YOL159C | CSS3 | Condition Specific Secretion | UP | UP | 1.11925E-11 | 2.61595E-09 | 2.61595E-09 |
| YFR010W | UBP6 | UBiquitin-specific Protease | DOWN | DOWN | 2.74899E-09 | 2.38907E-10 | 2.74899E-09 |
| YHR005C | GPA1 | G Protein Alpha subunit | DOWN | DOWN | 3.20669E-09 | 4.03407E-13 | 3.20669E-09 |
| YPL158C | AIM44 | Altered Inheritance rate of Mitochondria | DOWN | DOWN | 5.06344E-09 | 2.15945E-11 | 5.06344E-09 |
| YCL063W | VAC17 | VACuole related | DOWN | DOWN | 1.76892E-11 | 6.39072E-09 | 6.39072E-09 |
| YDR026C | NSI1 | NTS1 SIlencing protein 1 | UP | UP | 1.39263E-08 | 1.06728E-08 | 1.39263E-08 |
| YPL028W | ERG10 | ERGosterol biosynthesis | DOWN | DOWN | 3.05442E-11 | 2.17781E-08 | 2.17781E-08 |
| YJL157C | FAR1 | Factor ARrest | DOWN | DOWN | 2.69893E-08 | 7.4553E-20 | 2.69893E-08 |
| YCR100C | EMA35 | Efficient Mitochondria targeting-Associated protein | UP | UP | 2.74221E-08 | 1.60999E-23 | 2.74221E-08 |
| YIR001C | SGN1 | Slower Growth on Non-fermentable carbon sources | UP | UP | 2.69036E-22 | 2.89447E-08 | 2.89447E-08 |
| YPL061W | ALD6 | ALdehyde Dehydrogenase | DOWN | DOWN | 6.39404E-08 | 4.82936E-50 | 6.39404E-08 |
| YDR420W | HKR1 | Hansenula mrakii Killer toxin Resistant | UP | UP | 7.26211E-08 | 1.42845E-08 | 7.26211E-08 |
| YCR099C |  |  | UP | UP | 8.47517E-08 | 6.01232E-31 | 8.47517E-08 |
| YGR113W | DAM1 | Duo1 And Mps1 interacting | DOWN | DOWN | 6.07721E-08 | 8.77564E-08 | 8.77564E-08 |
| YJL130C | URA2 | URAcil requiring | UP | UP | 1.75681E-07 | 1.08742E-37 | 1.75681E-07 |
| YBR157C | ICS2 | Increased Copper Sensitivity | DOWN | DOWN | 1.00129E-10 | 1.8919E-07 | 1.8919E-07 |
| YOL104C | NDJ1 | NonDisJunction | UP | DOWN | 3.21835E-07 | 3.78123E-08 | 3.21835E-07 |
| YPR195C |  |  | UP | UP | 3.26211E-07 | 6.8817E-10 | 3.26211E-07 |
| YCR101C |  |  | UP | UP | 5.18181E-07 | 2.79364E-13 | 5.18181E-07 |
| YKR039W | GAP1 | General Amino acid Permease | DOWN | UP | 5.55552E-07 | 7.51295E-19 | 5.55552E-07 |
| YOR071C | NRT1 | Nicotinamide Riboside Transporter | DOWN | DOWN | 7.47107E-07 | 4.42716E-07 | 7.47107E-07 |
| YDL210W | UGA4 | Utilization of GAba | DOWN | UP | 9.92547E-07 | 8.60214E-16 | 9.92547E-07 |
| YER077C | MRX1 | Mitochondrial oRganization of gene eXpression (MIOREX) | UP | UP | 1.04613E-06 | 8.39699E-07 | 1.04613E-06 |
| YBR239C | ERT1 | Ethanol Regulated Transcription factor | UP | UP | 1.01651E-08 | 1.26521E-06 | 1.26521E-06 |
| YML126C | ERG13 | ERGosterol biosynthesis | DOWN | DOWN | 1.53884E-06 | 1.82616E-11 | 1.53884E-06 |
| YJR052W | RAD7 | RADiation sensitive | UP | UP | 1.21606E-11 | 1.74749E-06 | 1.74749E-06 |
| YNL156C | NSG2 |  | DOWN | DOWN | 2.1287E-06 | 2.17278E-09 | 2.1287E-06 |
| YGR044C | RME1 | Regulator of MEiosis | DOWN | DOWN | 3.10502E-24 | 2.71779E-06 | 2.71779E-06 |
| YLL029W | FRA1 | Fe Repressor of Activation | UP | UP | 8.22391E-09 | 3.03981E-06 | 3.03981E-06 |
| YDR351W | SBE2 | Suppressor of BEm4 | UP | UP | 5.07685E-16 | 3.50203E-06 | 3.50203E-06 |
| YBR291C | CTP1 | Citrate Transport Protein | UP | UP | 7.56776E-07 | 3.56624E-06 | 3.56624E-06 |
| YML085C | TUB1 | TUBulin | DOWN | DOWN | 5.38113E-07 | 3.87249E-06 | 3.87249E-06 |
| YPL175W | SPT14 | SuPpressor of Ty | UP | UP | 1.22303E-28 | 6.10704E-06 | 6.10704E-06 |
| YIR028W | DAL4 | Degradation of Allantoin | UP | UP | 6.16871E-06 | 7.20279E-08 | 6.16871E-06 |
| YGR040W | KSS1 | Kinase Suppressor of Sst2 mutations | UP | DOWN | 9.68607E-11 | 6.34154E-06 | 6.34154E-06 |
| YMR182C | RGM1 |  | UP | DOWN | 2.35727E-06 | 7.45834E-06 | 7.45834E-06 |
| YDR155C | CPR1 | Cyclosporin A-sensitive Proline Rotamase | DOWN | DOWN | 1.44222E-18 | 7.9262E-06 | 7.9262E-06 |
