# Supplementary material for: Cell integrity limits ploidy in budding yeast
Source: G3 (Bethesda). 2025 Jan 13;15(2):jkae286. doi: 10.1093/g3journal/jkae286 (PMC11797008; doi:10.1093/g3journal/jkae286)
Supplement: jkae286_Supplementary_Data [file jkae286_supplementary_data.zip › Table_S3_G3-2024-405275.docx]

| **Strain Identifier** | **Genotype** | **Derived from** |
| --- | --- | --- |
| 2587 | *MatA ade2-1 leu2-3 ura3 trp1-1 his3-11, 15 can1-100 GAL psi+* | W303 |
| 41742 | *MatA swe1::kanMX cdc24-1 ade2-1 leu2-3 ura3 trp1-1 his3-11, 15 can1-100 GAL psi+* | Derived from G. Fink F528 (S288C) backcrossed 4 X to W303 |
| 41898 | *MatA cln3::LEU2 mad1::URA3 bub2::HIS3 ade2-1 leu2-3 ura3 trp1-1 his3-11, 15 can1-100 GAL psi+* | W303 |
| 41904 | *MatA CLN3-∆Cterm::NatMX swe1::kanMX cdc24-1 ade2-1 leu2-3 ura3 trp1-1 his3-11, 15 can1-100 GAL psi+* | Derived from G. Fink F528 (S288C) backcrossed 5 X to W303 |
| 41994 | *MatA mad1::URA3 bub2::HIS3 ade2-1 leu2-3 ura3 trp1-1 his3-11, 15 can1-100 GAL psi+* | W303 |
| 42001 | *Matα cdc20-1 swe1::kanMX cdc24-1 ade2-1 leu2-3 ura3 trp1-1 his3-11, 15 can1-100 GAL psi+* | W303 |
| 42010 | *MatA CLN3-∆Cterm::NatMX mad1::URA3 bub2::HIS3 ade2-1 leu2-3 ura3 trp1-1 his3-11, 15 can1-100 GAL psi+* | W303 |
