# Supplementary figures and images for: Cell integrity limits ploidy in budding yeast
Source: G3 (Bethesda). 2025 Jan 13;15(2):jkae286. doi: 10.1093/g3journal/jkae286 (PMC11797008; doi:10.1093/g3journal/jkae286)

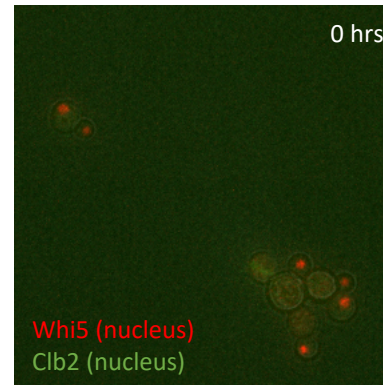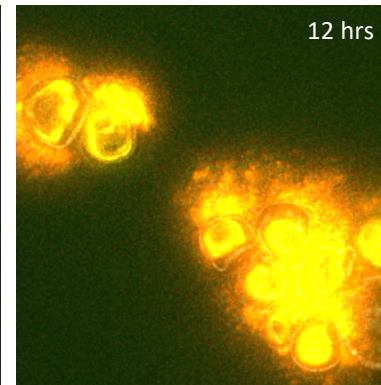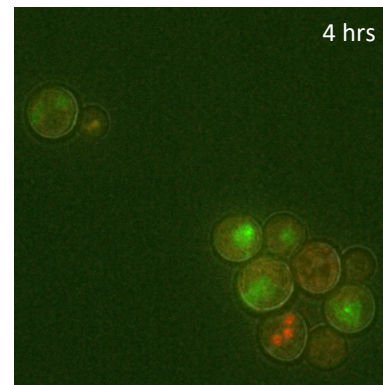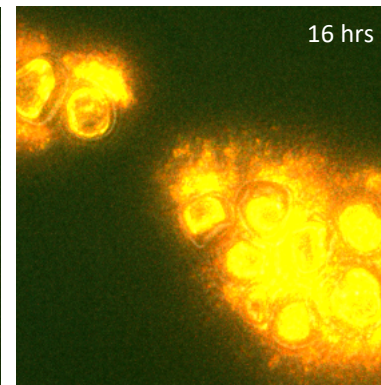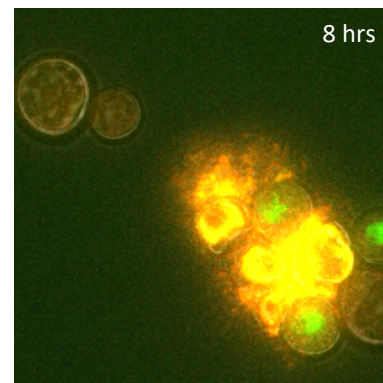

Supplement: jkae286_Supplementary_Data [file jkae286_supplementary_data.zip › Figure_S10_G3-2024-405275.pdf]

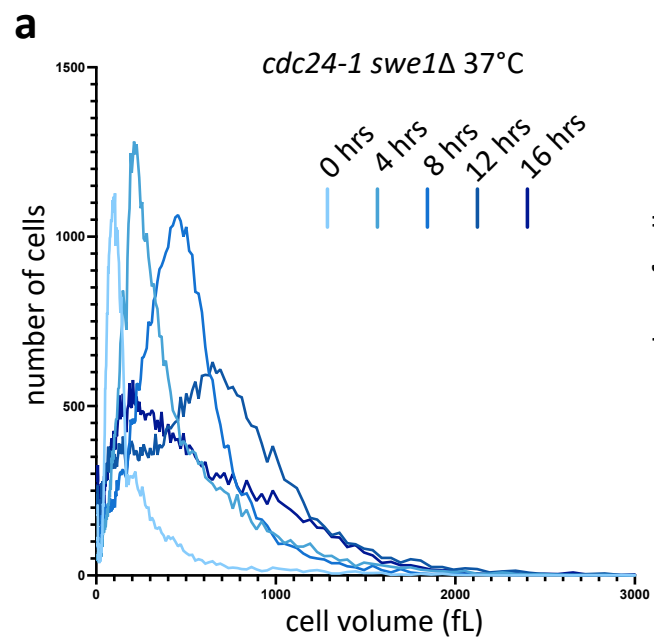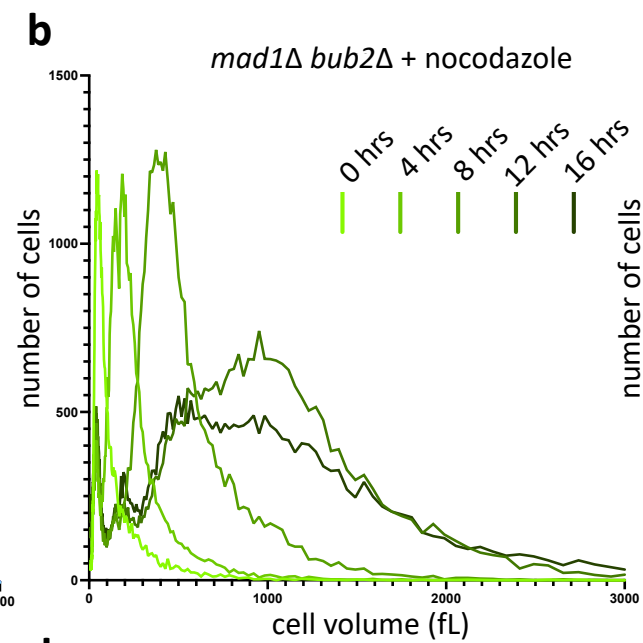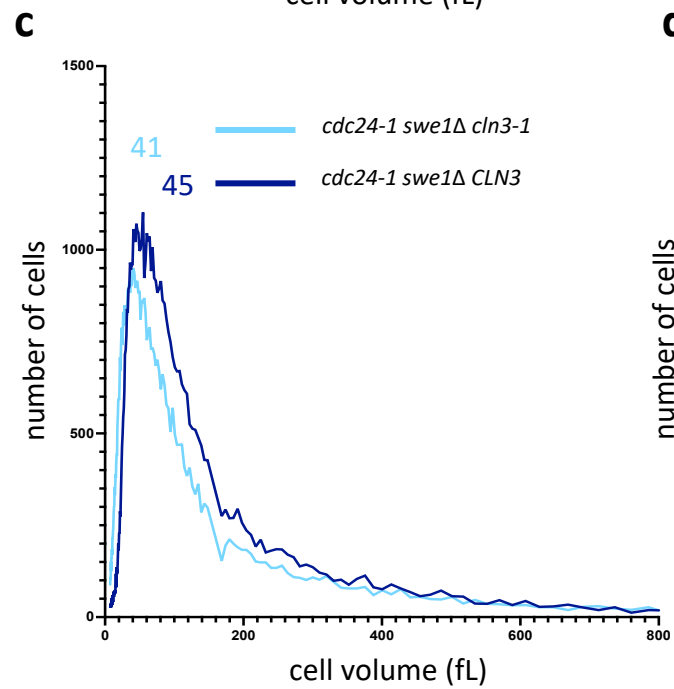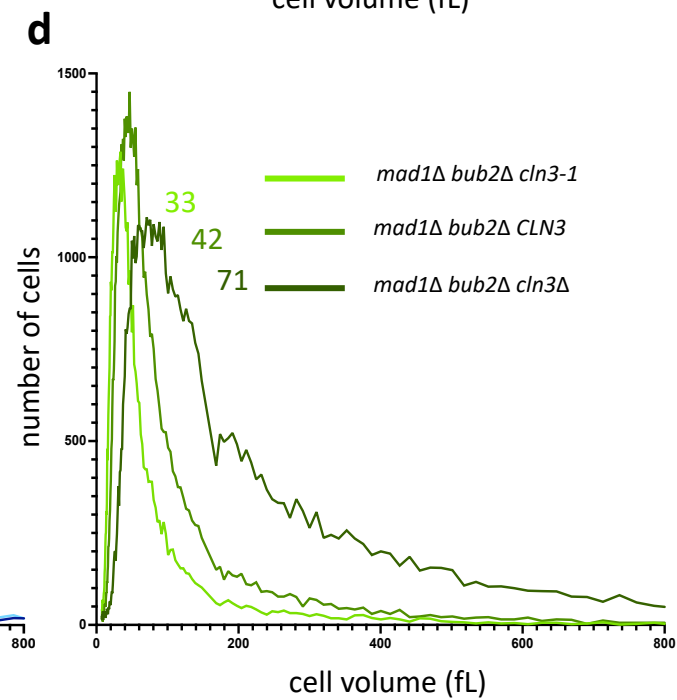

Supplement: jkae286_Supplementary_Data [file jkae286_supplementary_data.zip › Figure_S1_G3-2024-405275.pdf]

**a**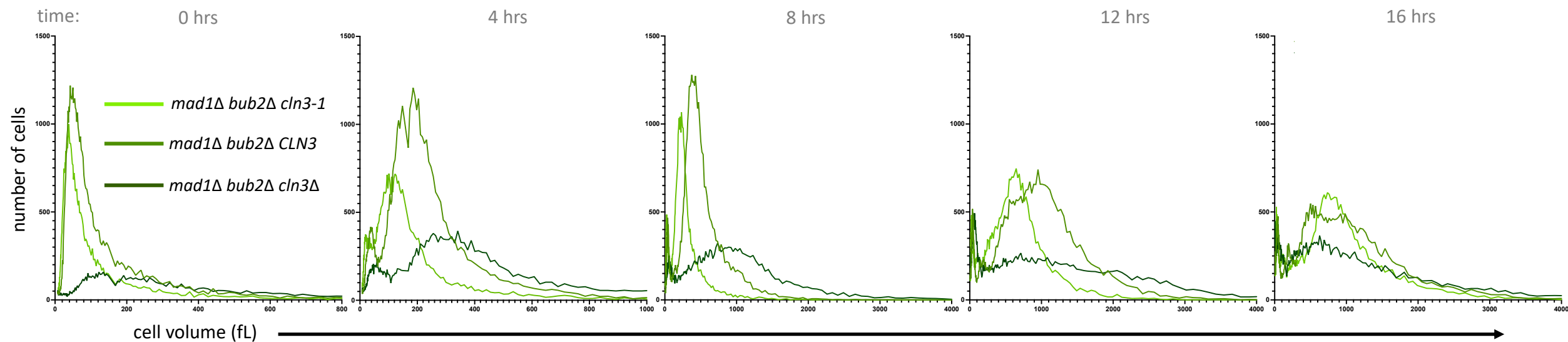**b**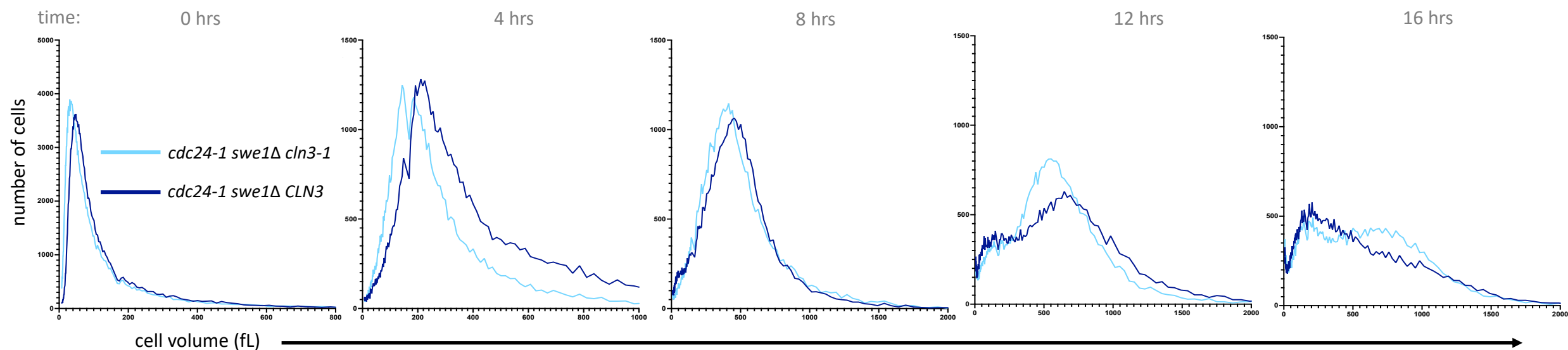

Supplement: jkae286_Supplementary_Data [file jkae286_supplementary_data.zip › Figure_S2_G3-2024-405275.pdf]

**a***mad1Δ bub2Δ* experiment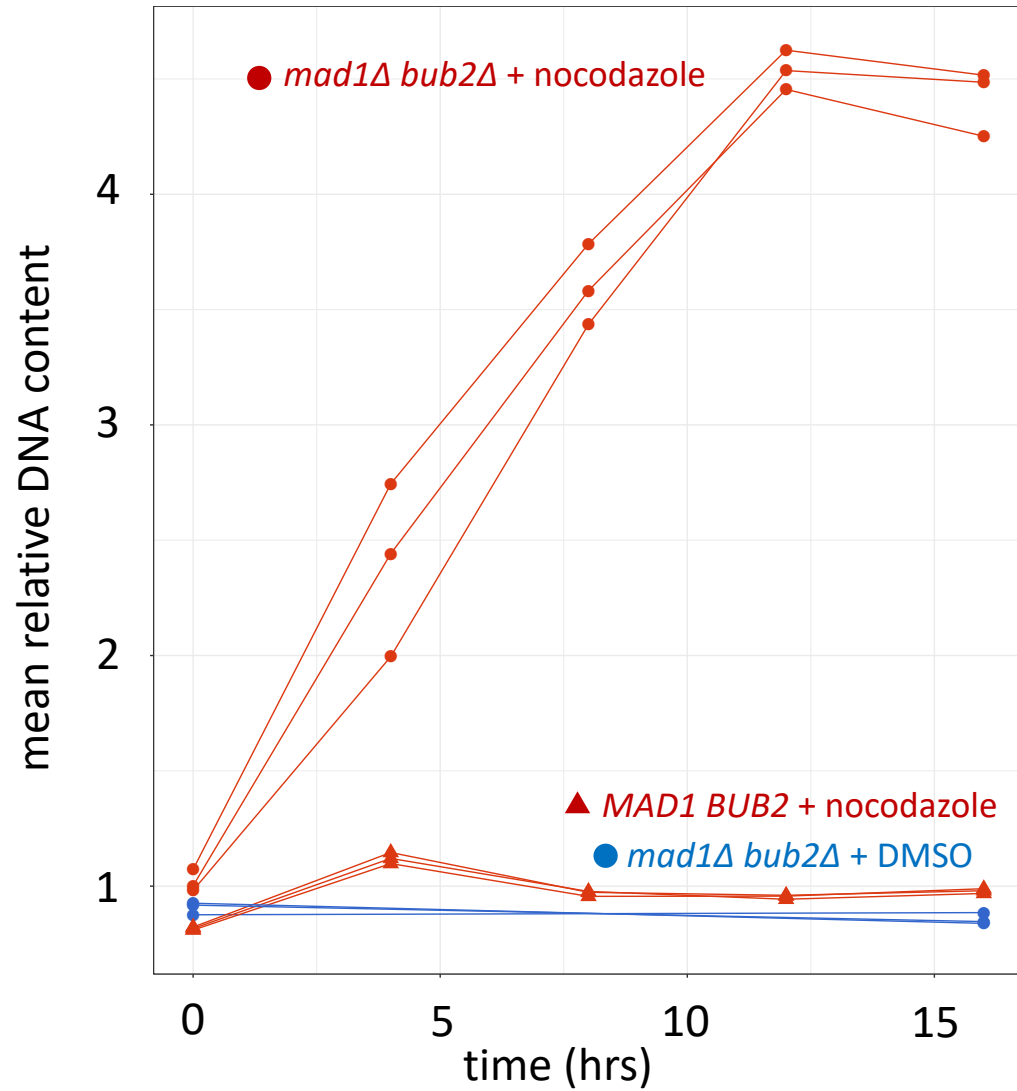**b***cdc24-1 swe1Δ* experiment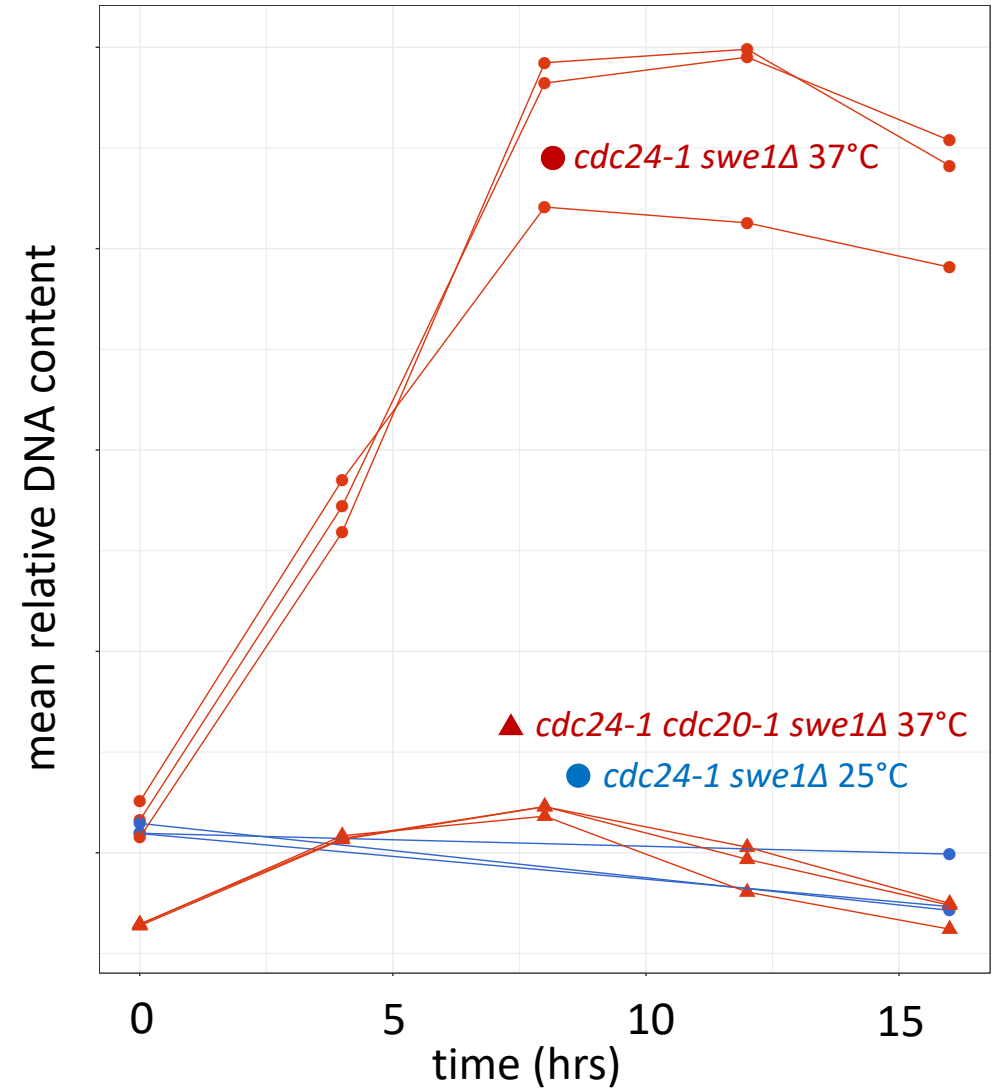

Supplement: jkae286_Supplementary_Data [file jkae286_supplementary_data.zip › Figure_S3_G3-2024-405275.pdf]

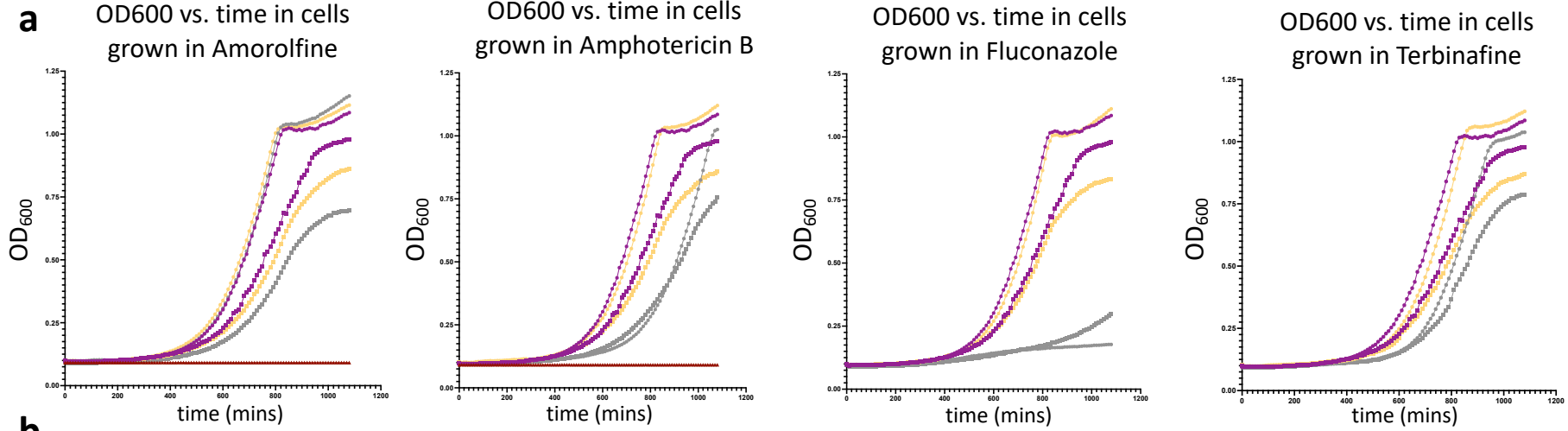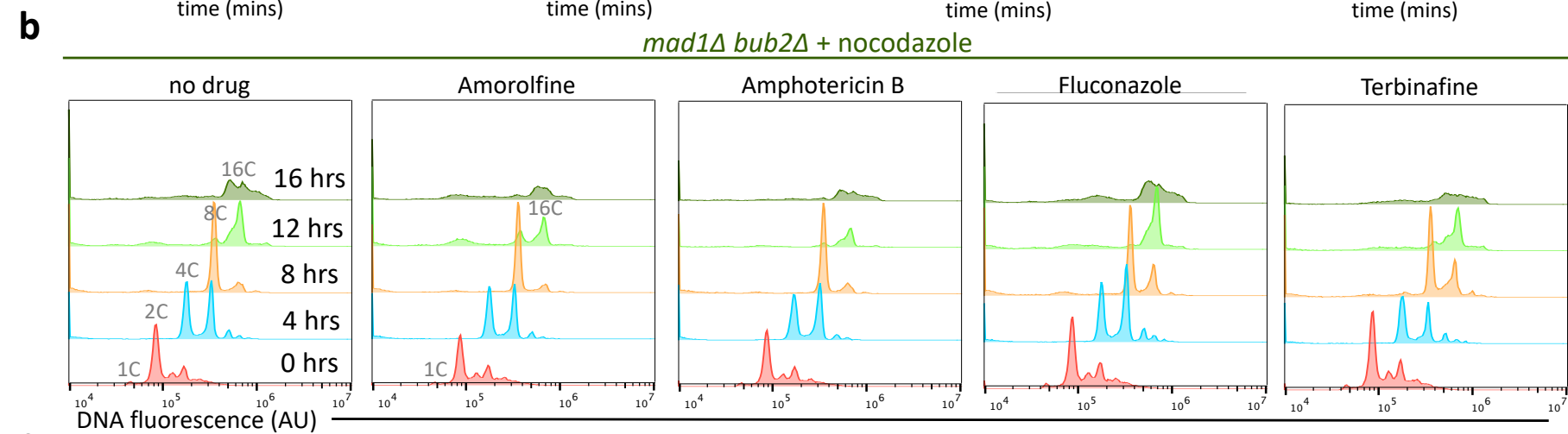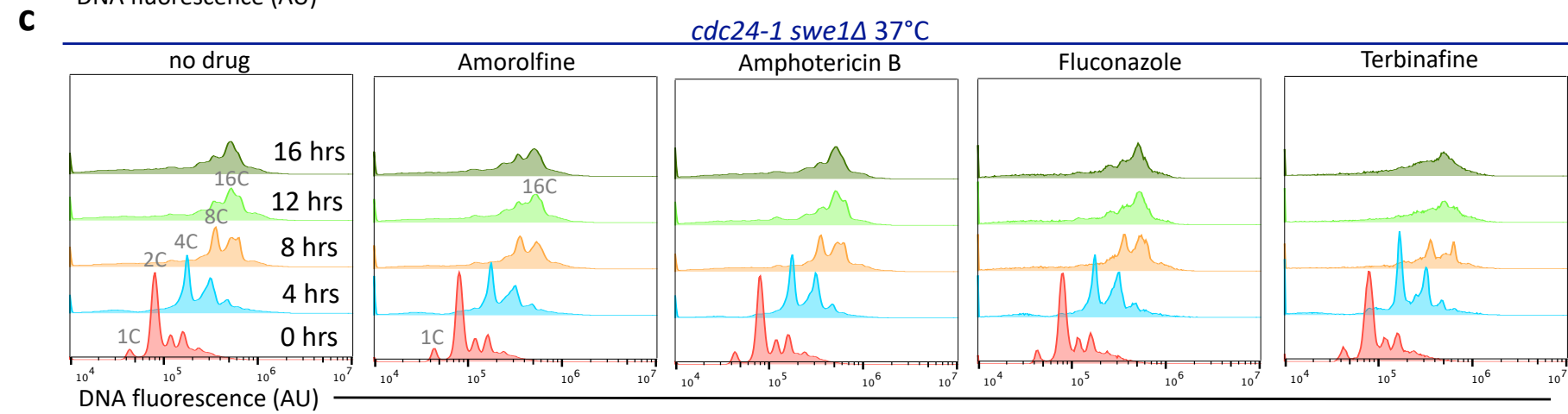

Supplement: jkae286_Supplementary_Data [file jkae286_supplementary_data.zip › Figure_S4_G3-2024-405275.pdf]

**a**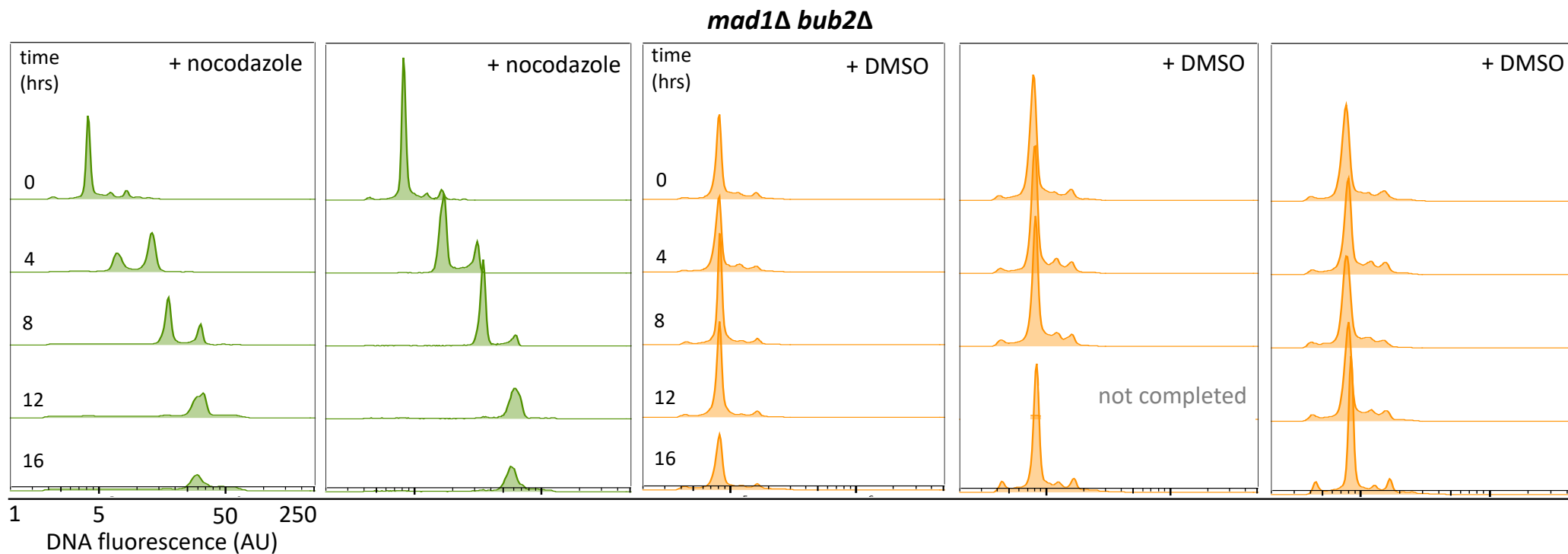**b**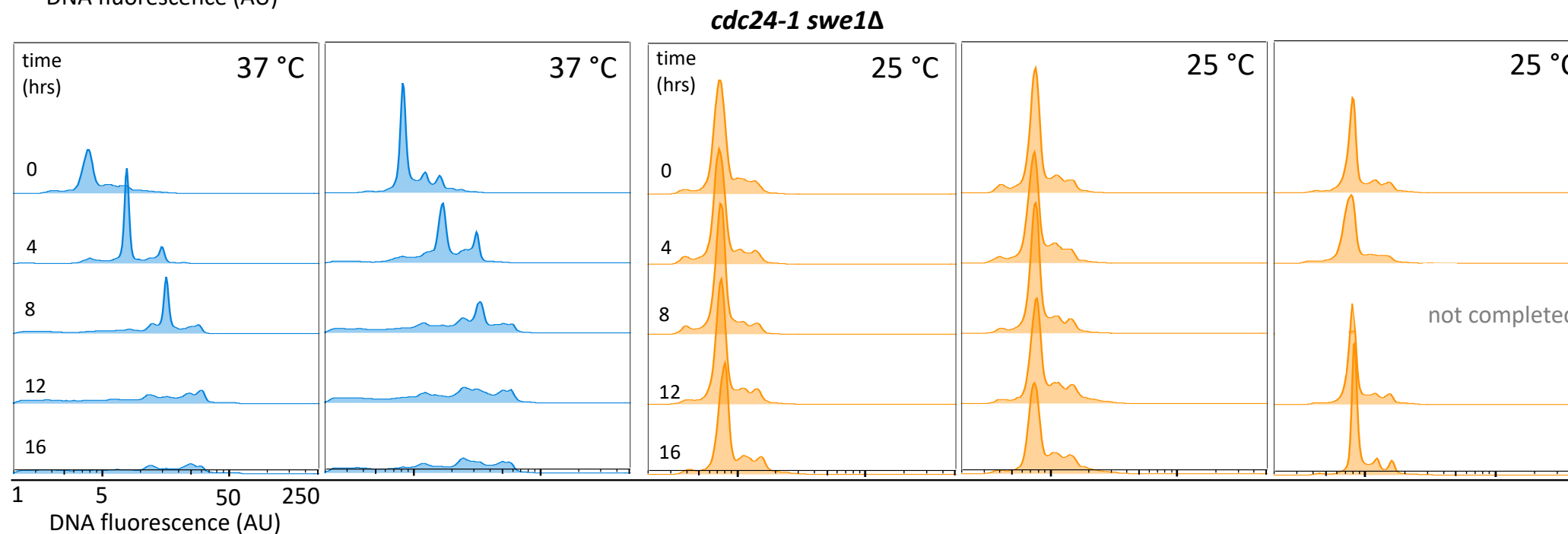

Supplement: jkae286_Supplementary_Data [file jkae286_supplementary_data.zip › Figure_S5_G3-2024-405275.pdf]

**a**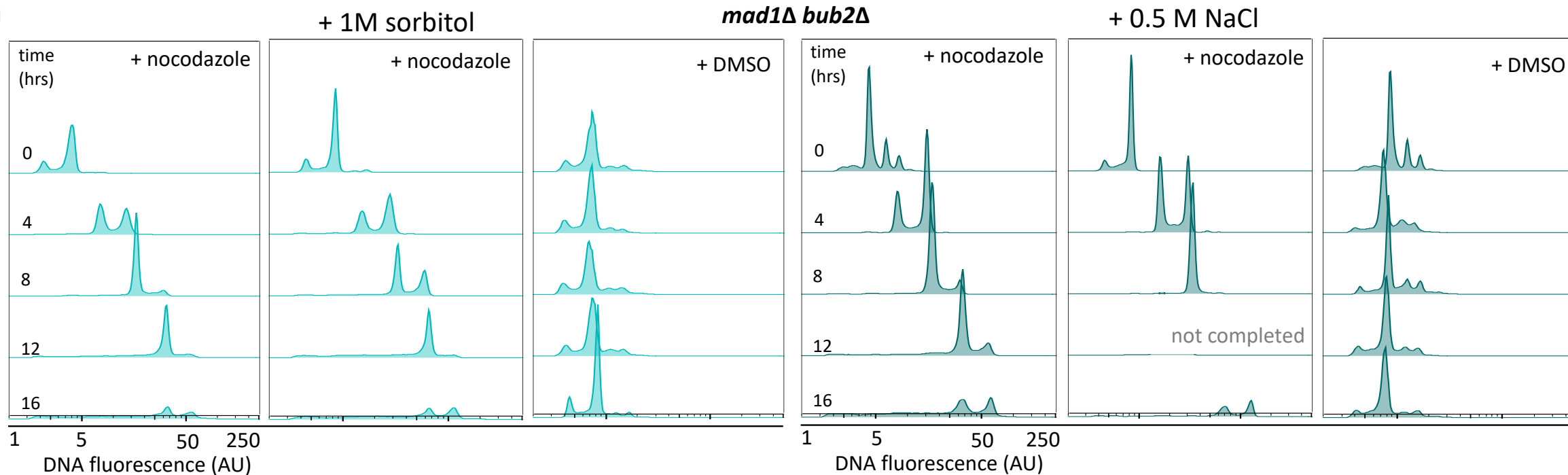**b**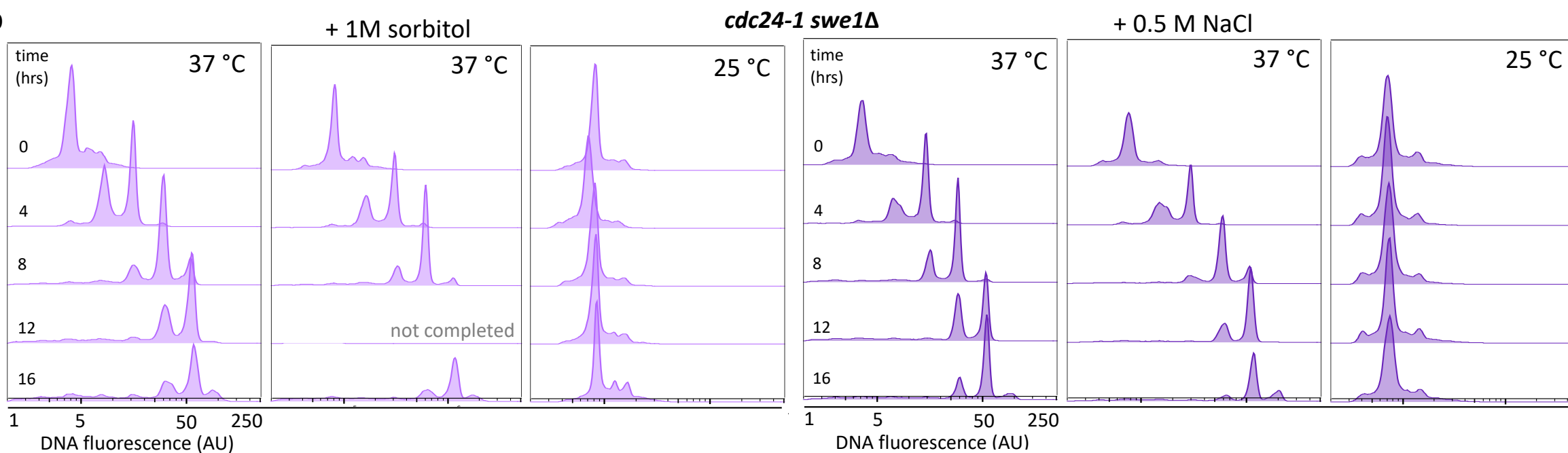

Supplement: jkae286_Supplementary_Data [file jkae286_supplementary_data.zip › Figure_S6_G3-2024-405275.pdf]

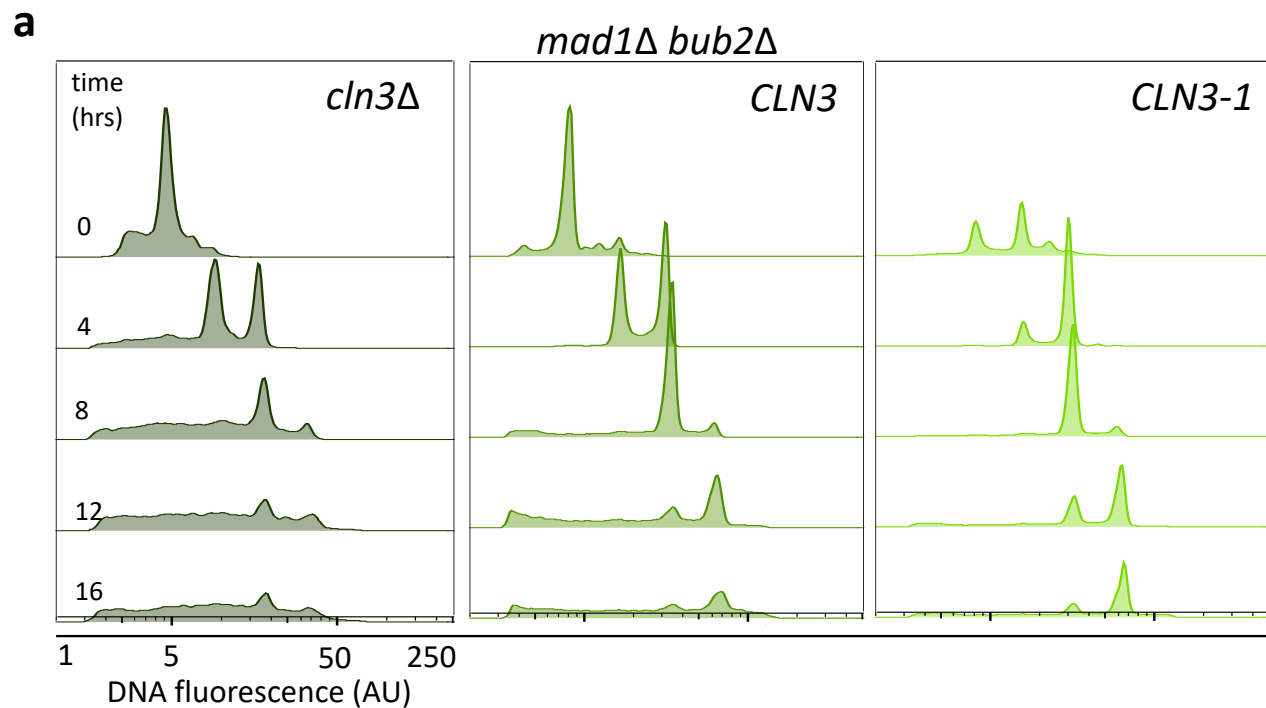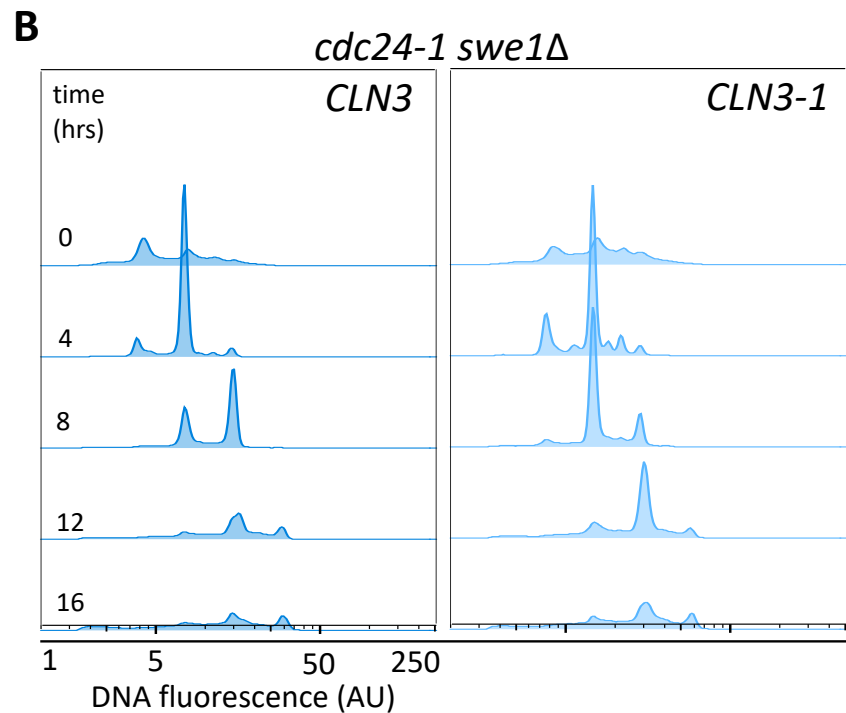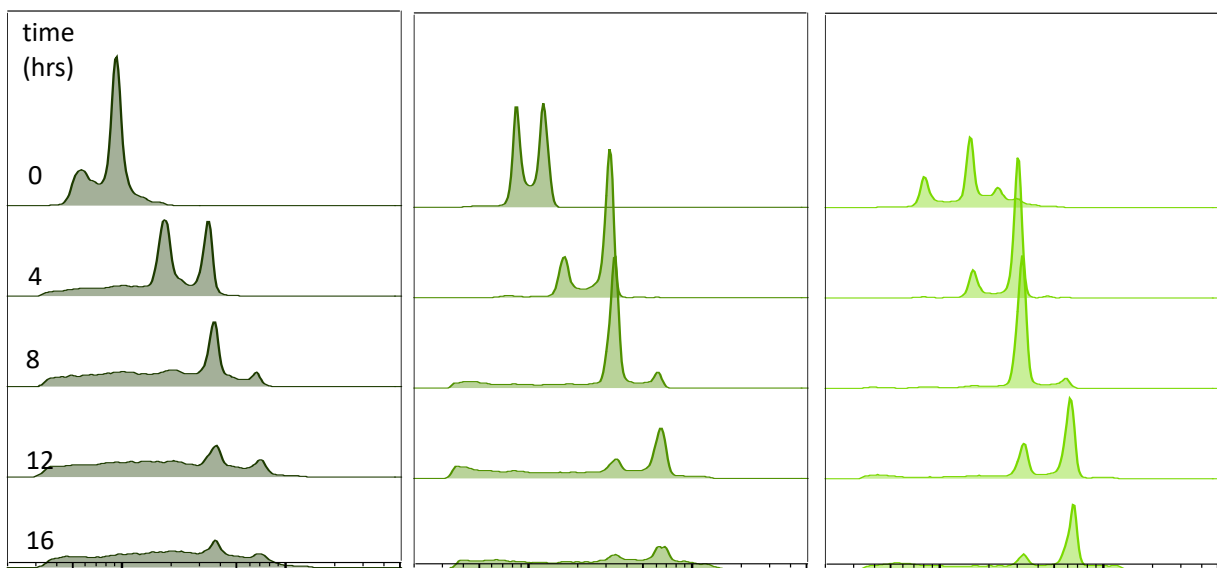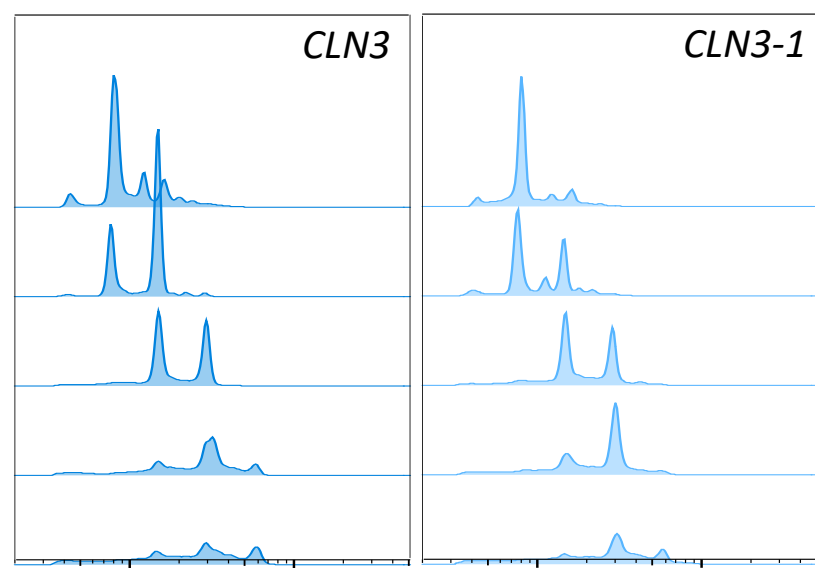

Supplement: jkae286_Supplementary_Data [file jkae286_supplementary_data.zip › Figure_S7_G3-2024-405275.pdf]

**a**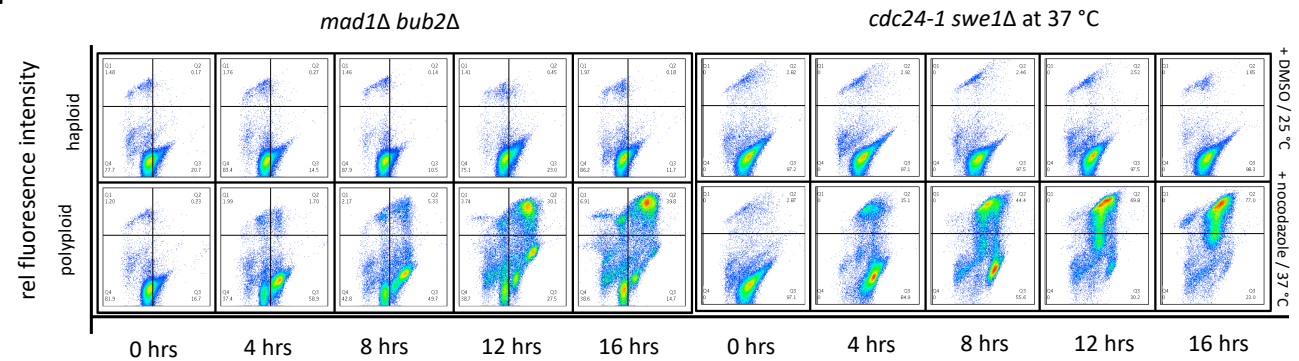**b**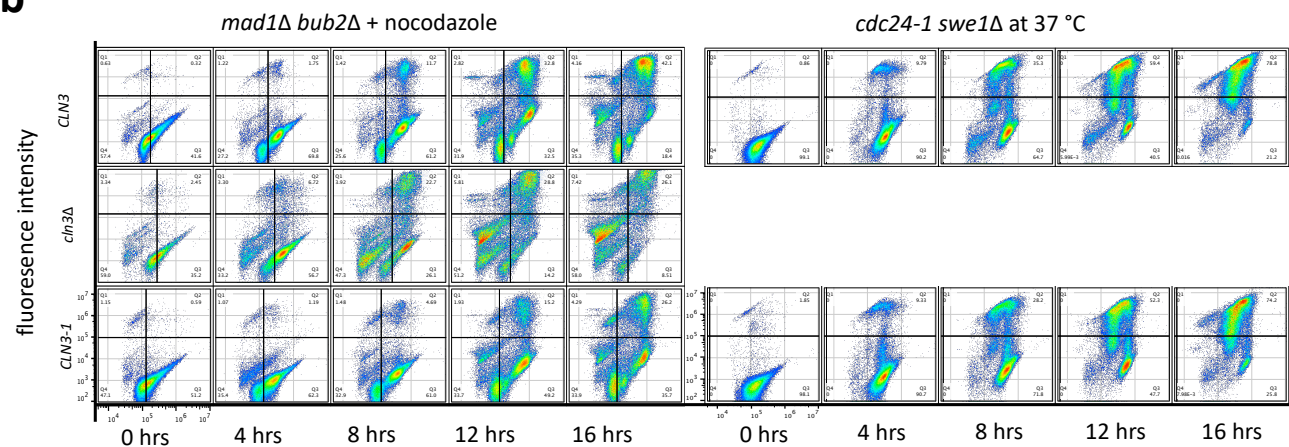**c**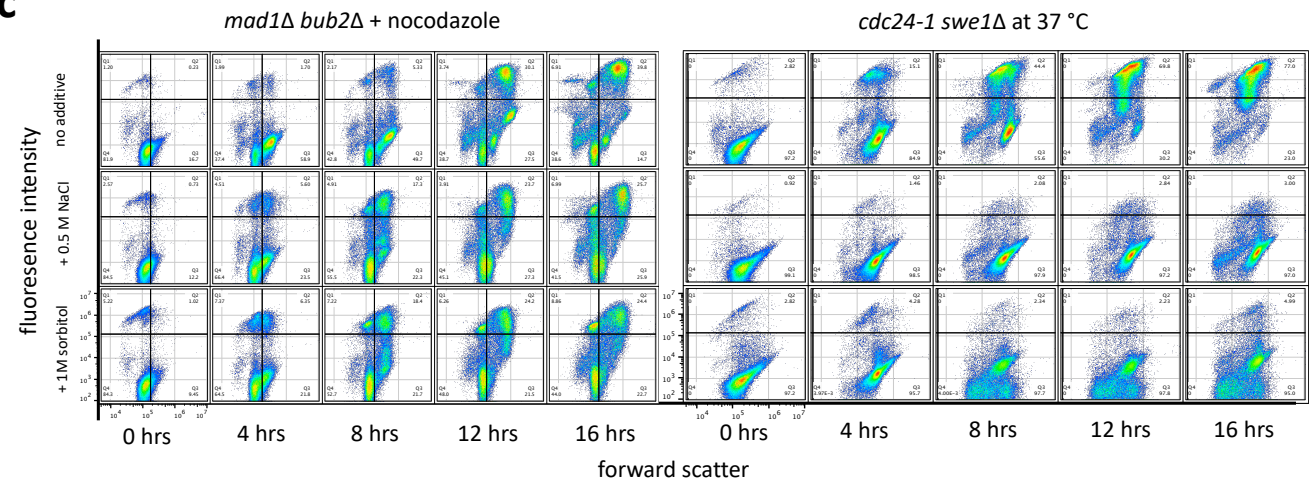

Supplement: jkae286_Supplementary_Data [file jkae286_supplementary_data.zip › Figure_S8_G3-2024-405275.pdf]

**a**normalized log<sub>2</sub>FC in gene expression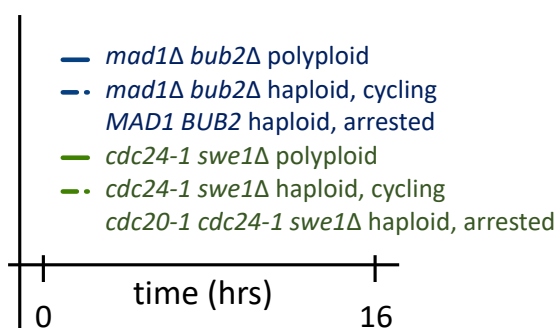**b**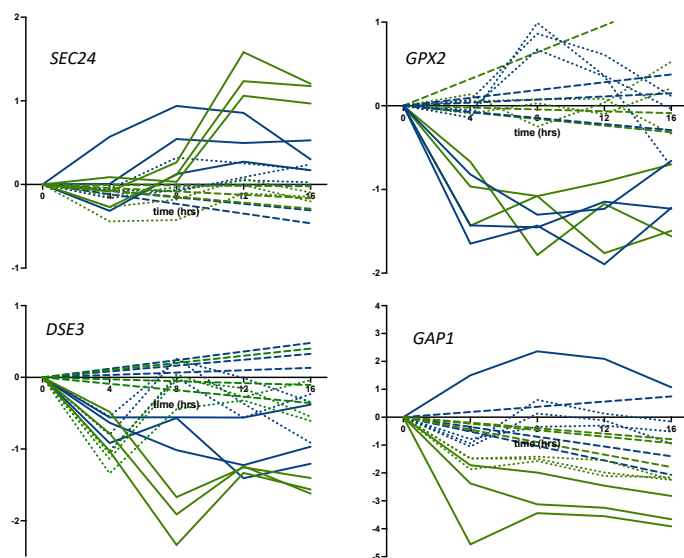**c**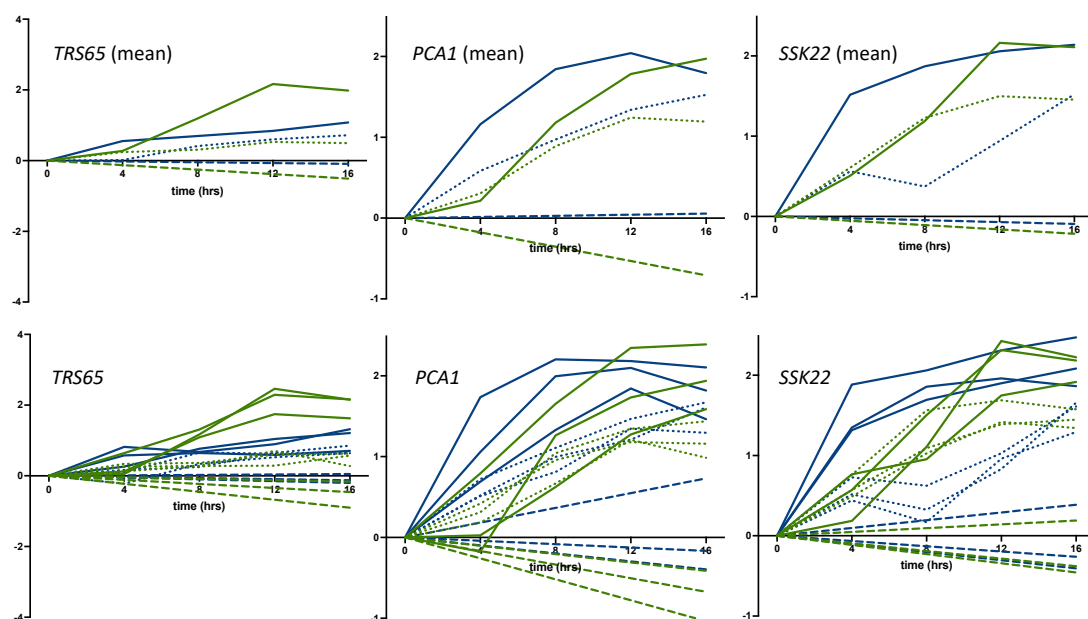**d**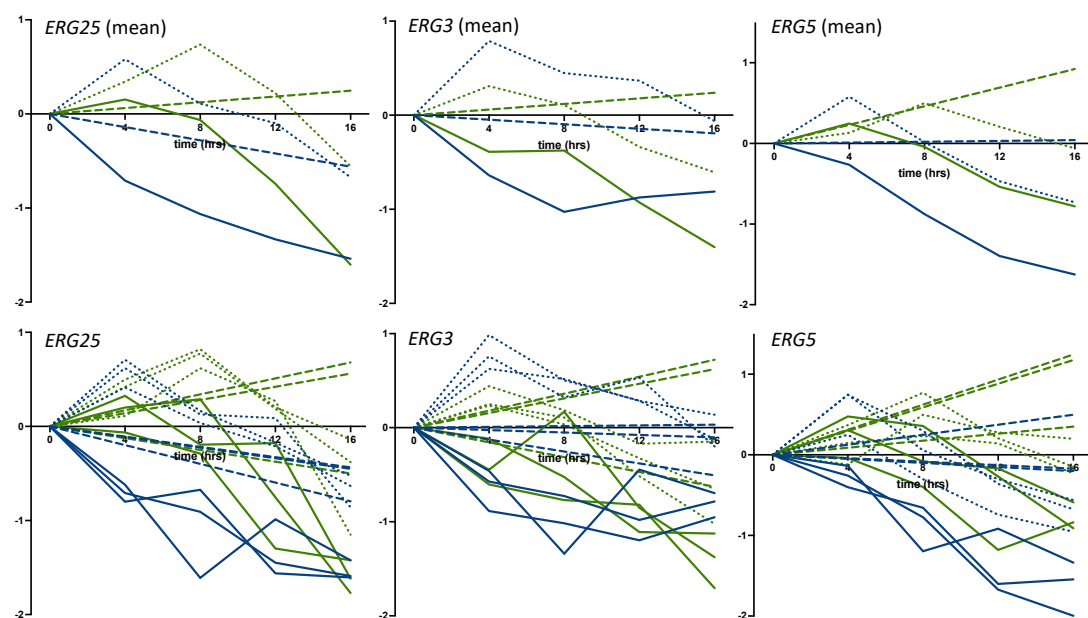

Supplement: jkae286_Supplementary_Data [file jkae286_supplementary_data.zip › Figure_S9_G3-2024-405275.pdf]
